# Dissecting the expression relationships between RNA-binding proteins and their cognate targets in eukaryotic post-transcriptional regulatory networks

**DOI:** 10.1038/srep25711

**Published:** 2016-05-10

**Authors:** Sneha Nishtala, Yaseswini Neelamraju, Sarath Chandra Janga

**Affiliations:** 1 Department of Bio Health Informatics, School of Informatics and Computing, Indiana University Purdue University, 719 Indiana Ave Ste 319, Walker Plaza Building, Indianapolis, Indiana 46202, USA; 2 Centre for Computational Biology and Bioinformatics, Indiana University School of Medicine, 5021 Health Information and Translational Sciences (HITS), 410 West 10th Street, Indianapolis, Indiana, 46202, USA; 3 Department of Medical and Molecular Genetics, Indiana University School of Medicine, Medical Research and Library Building, 975 West Walnut Street, Indianapolis, Indiana, 46202, USA

## Abstract

RNA-binding proteins (RBPs) are pivotal in orchestrating several steps in the metabolism of RNA in eukaryotes thereby controlling an extensive network of RBP-RNA interactions. Here, we employed CLIP (cross-linking immunoprecipitation)-seq datasets for 60 human RBPs and RIP-ChIP (RNP immunoprecipitation-microarray) data for 69 yeast RBPs to construct a network of genome-wide RBP- target RNA interactions for each RBP. We show in humans that majority (~78%) of the RBPs are strongly associated with their target transcripts at transcript level while ~95% of the studied RBPs were also found to be strongly associated with expression levels of target transcripts when protein expression levels of RBPs were employed. At transcript level, RBP - RNA interaction data for the yeast genome, exhibited a strong association for 63% of the RBPs, confirming the association to be conserved across large phylogenetic distances. Analysis to uncover the features contributing to these associations revealed the number of target transcripts and length of the selected protein-coding transcript of an RBP at the transcript level while intensity of the CLIP signal, number of RNA-Binding domains, location of the binding site on the transcript, to be significant at the protein level. Our analysis will contribute to improved modelling and prediction of post-transcriptional networks.

Progress in proteomics together with other omics technologies have now convincingly shown the existence of an additional and perhaps more important gene regulatory layer in cellular networks, which acts in concert with other layers of regulation to control gene expression and translation in a highly coordinated complex system defined as post-transcriptional regulatory network. For instance, in one of the large-scale omics studies comparing transcriptome and proteome levels it was shown that ~30% of the variance in protein abundance in yeast cannot be explained by mRNA expression levels^1^. Comparison of the dynamic transcriptome and proteome profiles in yeast also revealed the presence of several classes of post-transcriptionally regulated proteins, accounting for more than 40% of the proteome^2^. In another study, a comparison of functional clusters inferred from transcriptome and translatome data in yeast revealed the presence of three groups of proteins: transcriptionally co-regulated proteins cluster together in transcriptome as well as translatome data and represent metabolic processes; post-transcriptionally co-regulated proteins cluster together only in translatome data and consist of RNA-binding, ribosomal and protein synthesis proteins; and dually co-regulated proteins have intermediate co-clustering characteristics and hence are likely regulated at both levels^3^. Increasing number of studies now suggest that the lack of mRNA-protein correlation in eukaryotic cells can be explained due to the post-transcriptional control mediated by several regulatory RNAs with the major protein players being RNA-binding proteins (RBPs)^4^,^5^. Recent studies also show that RNA-binding proteins (RBPs) which play a crucial role in the post-transcriptional regulation of gene expression^4^,^5^,^6^ themselves exhibit distinct expression dynamics in post-transcriptional regulatory networks^7^ and tend to bind functionally related mRNAs with most mRNAs bound by multiple RBPs, resulting in a complex network of post-transcriptional regulatory interactions^8^,^9^.

In prokaryotes, functionally related genes are often organized into operons to facilitate co-expression and to reduce expression fluctuation among the resulting protein products^10^. Indeed, coordinated regulation of functionally related genes by reducing their expression variation in a cell is important for the survival of organisms with limited resources and has been observed in eukaryotes as well^11^,^12^,^13^,^14^. Hence, it was proposed that posttranscriptional regulons in eukaryotes may play an equivalent role to operon structures in prokaryotes in coordinating the expression of their target genes during posttranscriptional regulation^15^,^16^,^17^. According to the RNA regulon theory, trans-acting factors like RBPs combinatorially regulate multiple mRNAs to achieve functionally coherent translation in the face of stochastic gene transcription^14^. This posttranscriptional regulation of genes is important in splicing, transport, localization, translational control, stability and degradation of RNAs^9^,^14^,^18^. These various phases of RNA metabolism are regulated when the RBPs bind to the RNAs to form RNP complexes^4^,^9^. Therefore, the fate of RNA is dictated by the interaction of RNAs with the RBPs within the RNP complexes^6^,^19^. Various RBP mediated events have been well documented using expression profiles which are specific to tissues and conserved across different species^20^,^21^,^22^,^23^. With large amount of transcriptomic and proteomic data and a multitude of RBPs being identified, it has become possible to test if RBPs can direct the expression of their target transcripts using various flavors of RNA interactome datasets for RBPs in yeast and other model systems^24^,^25^,^26^. In particular, crosslinking immunoprecipitation (CLIP)-seq technology^27^ has proven to be a potent tool in the study and understanding of the transcriptomic *in-vivo* binding sites of RBPs at the single nucleotide level^28^. While experimental studies indicate that the function of RBPs on gene expression is complicated and sometimes can exhibit opposite trends depending on the growth condition, it is unclear whether RBPs can modulate expression levels of their target transcripts in humans and if there is an association between them in post-transcriptional regulatory networks^29^,^30^,^31^,^32^. Although a recent study suggests that RBPs are co-regulated with their target genes and plays an important role in coordinating their expression variation in yeast^25^, it is not clear how prevalent is this phenomenon and what factors contribute to such associations. In humans, numerous diseases have been linked to the defects in RBP function^33^,^34^,^35^. With many examples of RBPs being identified, it becomes feasible to test whether these post-transcriptional regulons can coordinate the expression of their target transcripts on a genome-wide level^25^ at least in model systems such as yeast and human with large-scale interactome data for multiple RBPs^8^,^36^,^37^, offering a unique opportunity to examine the regulatory relationships between RBPs and their target mRNAs^3^,^7^,^25^,^26^.

In this study, we map the CLIP binding sites of 60 RBPs on to the human genome to construct a RBP – RNA network. Further, we examined the correlation of each RBP’s expression at both transcript and protein levels with the target RNAs to see how these correlation patterns change. We then analyzed different factors impacting the change in expression patterns through a comprehensive two level analysis using different modelling techniques namely multivariate regression modelling, stepwise linear regression and Elastic net. We observed a higher level of association between the protein expressions of RBPs with their target transcripts compared to transcript level expression. Our results indicate that RBPs at both proteomic and transcriptomic levels play an important role in coordinating expression changes of the target RNAs and this can be explained by various factors governing the functions of these RBPs.

## Results

### Overview of the analysis

**Figure 1:**
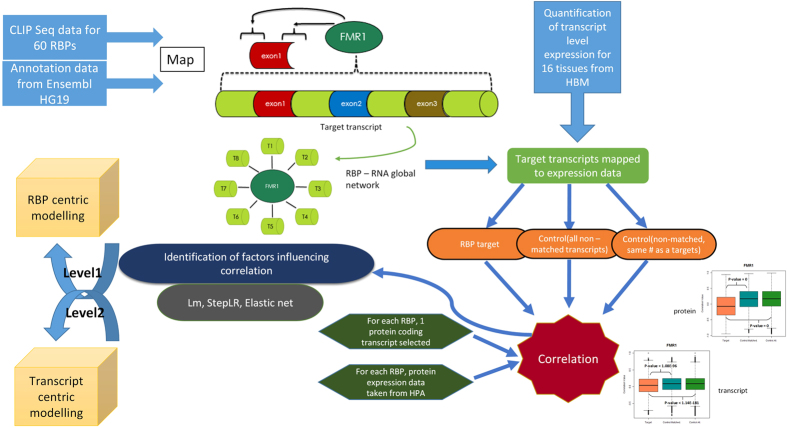
Flowchart summarizing the major steps involved in the construction and analysis of the human post-transcriptional regulatory network controlled by RBPs, employed in this study. Data required for the analysis was downloaded from CLIP DB^28^ and Ensembl^51^. The binding sites of each RBP were mapped to target transcripts such that, if the binding site of the RBP falls within the 300 bps flanking regions and 300 bps upstream regions of at least one of its annotated exonic start or end coordinates, its corresponding transcript would be considered a target transcript of the RBP. A global network was created for each RBP. The target transcripts were mapped on to the RNA-seq expression data from the Human Body Map (HBM)^52^,^53^,^54^ and three categories of transcripts were constructed based on whether a transcript group is targeted by an RBP or not. One protein coding transcript for each RBP with highest mean expression level across all 16 tissues was chosen and spearman correlation was calculated with transcripts from each of the three categories of transcripts. Similarly, correlations between protein expression levels of RBPs and their target/non-target transcripts expression levels from corresponding matched RNA-seq samples were calculated using protein expression data downloaded from Human Protein Atlas (HPA)^55^. Different patterns in associations between the RBPs and the three categories of transcripts were identified and classified. To explain the observed associations, three different feature selection/reduction methods were employed at two different levels, namely – RBP centric and transcript centric level.

As shown in Fig. 1, we downloaded CLIP data for 60 human RBPs from the CLIPdb database and used hg19 annotations from Ensembl database to build post-transcriptional regulatory networks linking RBPs to their target transcripts for each RBP (see Materials and Methods and Table 1). As discussed in Materials and Methods, we mapped the binding sites of each RBP to 300 bps upstream and downstream flanking regions of each exon and considered its corresponding transcript to be a target of the RBP if the binding sites map on these regions of the exons (Fig. 1). This allowed us to construct a genome-wide network of RBP-RNA interactions linking RBPs to their target transcripts in the human genome as summarized in Table 1. The target transcript annotations were compared with the quantified transcript level expression data across 16 human tissues from the Human Body Map (HBM) project and were divided into three groups of transcripts for each RBP as described in Materials and Methods using ad hoc python scripts (Fig. 1). For each RBP, both the transcript level of one protein coding transcript and its protein expression data from Human Protein Atlas (HPA) were independently used for correlation analysis with its target transcripts as described in Materials and Methods. Similar approach was employed for analyzing the associations between RBPs and their target transcripts in the constructed post-transcriptional regulatory network of the yeast genome (see Materials and Methods). To understand the different factors influencing the observed correlation patterns at both the transcript and protein levels in the human genome, we undertook a comprehensive modelling approach using three different feature selection/reduction methods at two different levels – RBP centric and transcript centric level by considering the different factors listed in Tables 2 and 3. A discussion of the selected features is presented in the Materials and Methods and the respective results section.

### Majority of the RBPs exhibit significant association with their target transcripts at the transcript level

**Figure 2:**
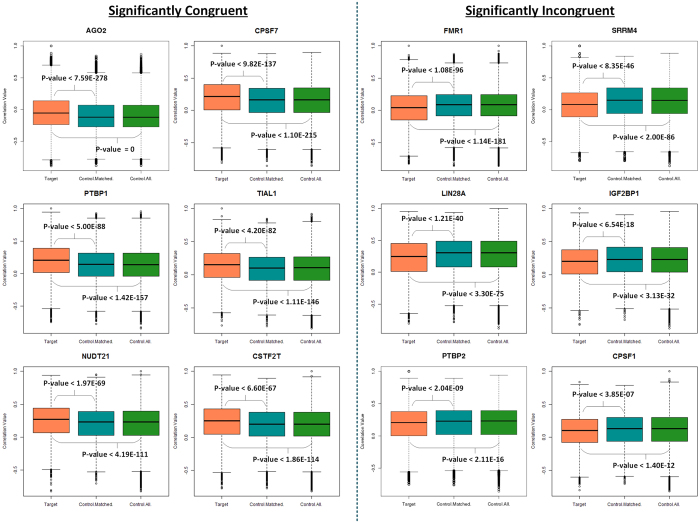
Selected set of six human RBPs each belonging to the significantly congruent and incongruent categories, when only transcriptome data was used for computing the correlations between RBPs and their target/non-target transcripts. Boxplots showing the distribution of correlation coefficients between RBPs and transcripts belonging to the three categories, red: protein coding transcript of RBP correlated with its target transcripts, blue: protein coding transcript of RBP correlated with the same number of random non-targeted transcripts as the number of target transcripts in the post-transcriptional regulatory network of an RBP, green: protein coding transcript of RBP correlated with all the non-targeted transcripts.

To understand how RBPs are associated with their targets, we correlated the expression of one of the protein coding transcripts (with the highest mean expression level across all the tissues) of each RBP with the expression of the target transcripts across 16 human tissues and compared it with control set of transcripts (all transcripts which do not belong to the class of target transcripts). To address the issue of the size of the control set, we also randomly sampled the control set of transcripts by sampling 100 times to extract each time the same number of randomly selected transcripts as the number of target transcripts, which is referred to as the control-matched set. As a result of computing the spearman correlations between a RBP transcript and its target as well as non-target (control) transcripts, RBPs were divided into three classes based on comparing the distribution of correlation coefficients for targets *versus* control associations; 1) Significantly Congruent (SC) : RBPs would belong to this class if the distribution of RBP – target correlation coefficients have their median correlation coefficient significantly higher than that seen in the control set of transcripts (Wilcoxon test, p < 0.05) 2) Significantly incongruent (SIC) : RBPs would belong to this class if the distribution of RBP – target correlation coefficients have their median correlation coefficient significantly lower than that seen in the control set of transcripts (Wilcoxon test, p < 0.05) 3) No Significant change (NSC) : If no significant change in the median correlation coefficient is observed between the targets and the control sets, those RBPs would belong to this class. RBPs grouped into these three classes are supported by robust set of p–values as illustrated in Supplementary Fig. 1. Figure 2 shows the six most significant SC and SIC RBPs at the transcript level. PTBP1 with PTB domain^38^,^39^, known to control pathways related to translational control and splicing and CSTF2T for mRNA – splicing^40^ are among the significant SC RBPs. FMR1 known to be important for translation control and documented to be implicated in several neurological disorders^41^ as well as LIN28A known for cardiac progenitor differentiation and translational control^42^ were found to be among the significant SIC RBPs. Supplementary Fig. 1 shows boxplots comparing the correlation coefficients for target *versus* control transcripts for all the 60 RBPs organized into SC (36 RBPs), SIC (11 RBPs) and NSC (13 RBPs) classes. Overall, we found that 78.33% of the RBPs comprised of SC and SIC classes, exhibited significant association with their targets at the transcript level, at a p-value threshold of 0.05.

### Significant fraction of the yeast RBPs also exhibit an association with their target transcripts revealing the conservation of expression coupling

**Figure 3:**
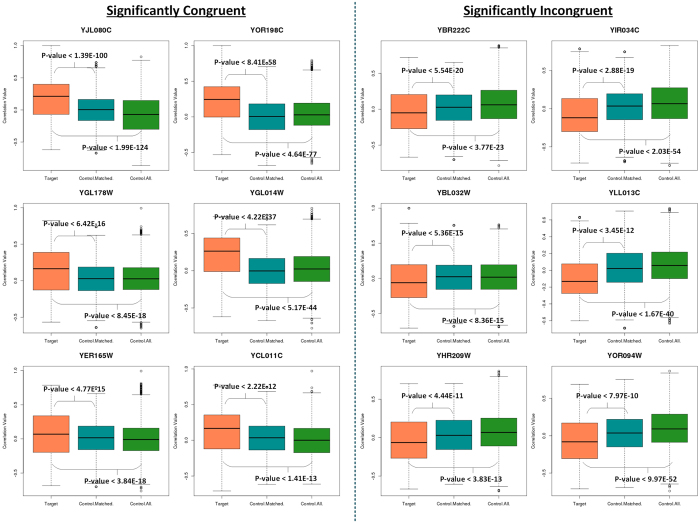
Selected set of six yeast RBPs each belonging to the significantly congruent and incongruent categories from the yeast post-transcriptional regulatory network of RBPs^43^. Boxplots showing the distribution of correlation coefficients between RBPs and transcripts belonging to the three categories, red: transcript expression of RBP correlated with its target transcripts, blue: transcript expression of RBP correlated with the same number of random non-targeted transcripts as the number of target transcripts in the post-transcriptional regulatory network of an RBP, green: transcript expression of RBP correlated with all the non-targeted transcripts.

To understand, whether our observation of finding RBP – target expression correlations to be significantly non-random, is generic and conserved across organisms, we analyzed the RBP – RNA network of the yeast genome using the same workflow (see Materials and Methods). Since humans and yeast are evolutionary distant, we hypothesized that the yeast genome would be an ideal model to show the generality of our observations across different species. The network used in this study^43^ comprises of 69 RBPs corresponding to 24,932 RBP – RNA interactions. On performing a similar analysis to that described for the human RBPs, we found comparable results in the yeast genome. In particular, the correlation patterns revealed that 63.08% (41/65) of the RBPs display an association (Wilcoxon test, p < 0.05) (Supplementary Fig. 2). Among the RBPs which exhibited a significantly higher/lower correlation coefficient compared to control transcripts, 58.54% (24) could be classified as SIC and 41.46% (17) as SC RBPs. Figure 3 shows the six most significant SC and SIC RBPs. YJL080C, commonly known as - SCP160 is important for mRNA metabolism in yeast^44^ and interestingly, in our analysis, it is shown to be one of the most significantly associated RBPs with its target mRNAs. Similarly, YIR034C or LYS1 is important for mRNA binding in yeast^45^,^46^ and is one among the highly associated SIC RBPs. Supplementary Fig. 2 shows all the 65 RBPs organized into SC, SIC and NSC classes.

### Most RBPs exhibit significant association with their target transcripts at the protein level

**Figure 4:**
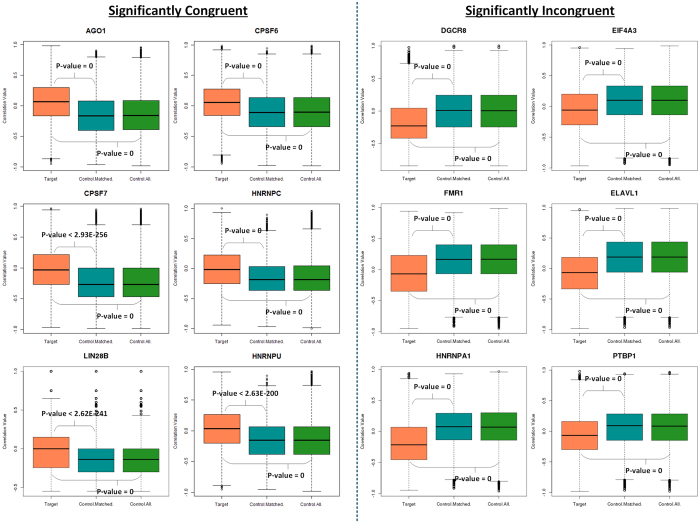
Selected set of six human RBPs each belonging to the significantly congruent and incongruent categories, when protein expression levels of RBPs and transcript levels of the target/non-targets was used for computing the correlations. Boxplots showing the distribution of correlation coefficients between RBPs and transcripts belonging to the three categories, red: protein expression level of an RBP correlated with its target transcripts, blue: protein expression level of an RBP correlated with the same number of random non-targeted transcripts as the number of target transcripts in the post-transcriptional regulatory network of an RBP, green: protein expression level of an RBP correlated with all the non-targeted transcripts.

While we found that RBPs show good degree of associations with their targets in both the human and yeast genomes when the transcript levels of RBPs are employed, protein expression levels of RBPs in matched tissues or experimental conditions is rather limited. However, recent genome-wide protein levels for multiple human tissues resulting from the Human Proteome Map facilitate addressing this question, albeit using limited number of samples (see Materials and Methods). After identifying and mapping equivalent RNA-seq and proteomic samples, we correlated the protein expression data across nine tissues for each RBP with the corresponding target as well as control transcripts’ expression levels from the RNA-seq dataset and organized the RBPs in to three classes – SC, SIC and NSC. Figure 4 shows six most significant SC and SIC RBPs at the protein level. Several members of CPSF and HNRNP family exhibited significant correlation with their targets often in different directions. Supplementary Fig. 3 shows all the 58 RBPs organized into the three different classes – SC (11 RBPs), SIC (44 RBPs) and NSC (3 RBPs). Note that two of the RBPs, RTCB and SRRM4 had no expression levels documented in the protein expression dataset and hence were not included in this analysis. Overall, we found that ~95% of the RBPs exhibited significant association with their target transcripts at the protein level (Wilcoxon test, p < 0.05). These results support the notion that the protein expression levels of most RBPs are strongly correlated with their target transcripts expression levels. Indeed, this association is far stronger than that observed at the transcript level of an RBP.

### Only a small fraction of the RBPs show similar patterns of associations with their targets at both the protein and transcript levels

**Figure 5:**
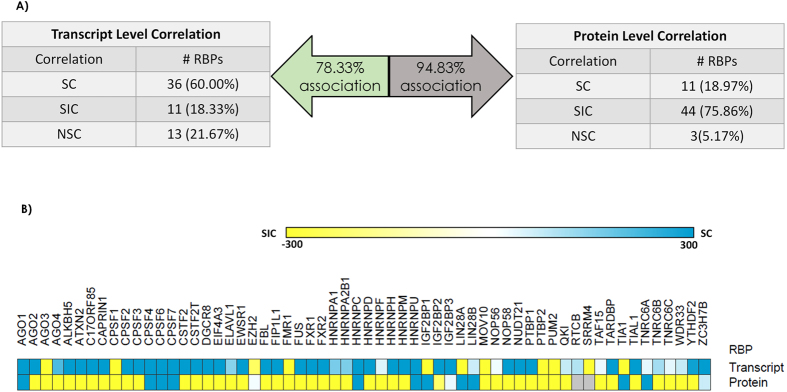
Summary of the various correlation patterns observed at the transcriptomic and proteome levels for human RBPs. (**A**) Distribution of RBPs into SC, SIC and NSC categories when the RNA and protein expression levels of the RBP respectively, are considered for computing the correlations. (**B**) Heatmap showing the significance (−log(p-value)) of the observed correlation compared to that seen in random non-targeted transcripts for various RBPs when the RNA and protein expression levels of an RBP are considered. P-values are computed using the Wilcoxon test comparing the distributions of correlation coefficients between targeted and 100 sets of non-targeted transcripts for each RBP. In the heatmap, blue color represents the SC RBPs and the yellow color represents the SIC RBPs.

To further understand and dissect our findings on RBP – target associations, we performed a comparative analysis of the outcomes for various human RBPs to see how these correlation patterns change within the transcript or protein levels and from the transcript to the protein levels of RBPs. Figure 5A summarizes the results of our analysis by showing the percentage of RBPs showing associations at the transcript and protein levels, number of RBPs falling into each of the three classes - SC, SIC and NSC, while Fig. 5B shows a heatmap showing the significance values (−log(p-value)) of the RBP – target associations compared to 100 matched control sets used in the previous sections. We find that at the transcript level 60% of the RBPs fall into the SC category while at the protein level, 18.97% fall into this category. Likewise, 18.33% RBPs fall into the SIC category at the transcript level while 75.86% RBPs fall into this category at the protein level.

Further, we observed that 12 RBPs exhibited similar trends at both the transcript and protein levels with six of them belonging to SC category and six belonging to the SIC category. To further understand the behavior of these 12 RBPs (sync RBPs) and how they are different compared to others (non-sync RBPs), we analyzed different network centrality measures using igraph^47^ package in R. This was achieved by constructing a protein interaction network for RBPs using two different sources - Biogrid databas^48^ and String database^49^ separately, to obtain an unbiased understanding of the differences in the network centrality measures irrespective of the dataset used. We found that closeness centrality for sync RBPs is higher than non-sync RBPs using interaction networks from both Biogrid and String databases (Wilcoxon test, p < 3.55E-08 and p < 1.80E-10 respectively) indicating that sync RBPs have shorter average path lengths to other proteins in the protein interaction network and hence must be well connected to other proteins (Supplementary Table 1). Using the interaction network from String database, we also found betweenness centrality to be different between the sync and non-sync groups (Wilcoxon test, p < 0.006). Therefore, we postulate that sync RBPs are likely functionally active and remain the same at the protein level too.

### Different set of features influence the correlation observed at the transcript and protein levels

**Figure 6:**
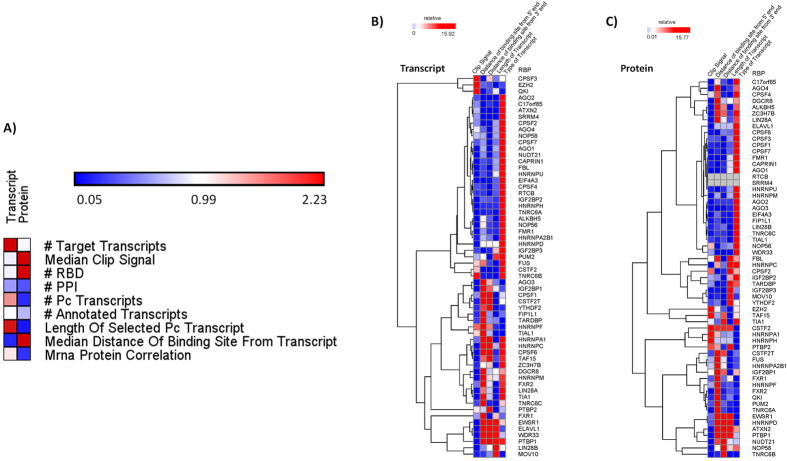
Heatmaps showing the significance of various features influencing correlation at the RBP and transcript centric levels. Heatmaps show the significance values (−log(p-value)) obtained by performing multivariate regression modelling to predict features influencing correlation at the (**A**) RBP level. (**B**,**C**) Transcript level when RNA and protein levels of RBPs were considered. Significance values for various features considered in this analysis are clustered hierarchically. Similar results were obtained using stepwise linear regression and elastic net regression modelling.

As listed in Table 2 and discussed in Materials and Methods, nine features were selected which we hypothesized to contribute to the observed correlation patterns between RBPs and their transcripts. We selected these features for RBP centric modelling because each of these features are likely contributing to the function or dynamics of an RBP, its influence on the target transcript or can be attributed to the strength of its binding signal on the target transcript in either a direct or indirect mode and hence would therefore be explanatory of the observed trend. We used three different feature selection approaches, multivariate regression, step wise linear regression and elastic net to identify a reproducible and robust set of important features. Figure 6a shows the significance (−log(p-value) for all the features tested, at the transcript and protein level plotted as a heatmap. We found that at the transcript level, the number of target transcripts, number of protein coding transcripts and the length of the selected protein coding transcript were the most important features while at the protein level, median clip signal, number of RNA binding domains and median distance of the binding site on the transcript as important features. Similar results were obtained using the elastic net framework. Supplementary Fig. 4 displays the important features obtained using this method.

### Type of the transcript and distance of the binding site from either side of the transcript prove to be important features at the transcript centric level

As listed in Table 3, five features were selected which we hypothesized to contribute to the observed correlation patterns between RBPs and their transcripts at the transcript level modelling (see Materials and Methods). Briefly, in transcript centric modelling, the response variable is the correlation coefficient between each RBP and its target transcript essentially enumerating all possible RBP-transcript pairs. As earlier, we used three different methods similar to the procedure described for the RBP centric modeling to uncover the robust set of features. Figure 6b,c show the significance (−log(p-value)) of the different features for the various RBPs using multivariate regression modeling. A similar figure without clustering is available as Supplementary Fig. 5, for easier reference. We found that transcript type is a significant feature for 66.67% and 63.80% of the RBPs at the transcript and protein levels respectively. There were a total of 56 transcript types that were available from Ensembl BioMart^50^ into which our target transcripts were classified. We therefore tried to understand which transcript type is more contributing to the response variable by inspecting the median correlation coefficient of each transcript type for each RBP showing transcript type as an important feature. We observed that interchangeably, protein coding transcript and processed transcript followed by miRNA and lncRNA were the important transcript types impacting the expression correlation. We also found that the distance of the binding site of the RBP from 3′ or 5′ end of the transcript were important factors for many RBPs.

## Discussion

In summary, we find that RBPs exhibit significant co-expression patterns with their target RNAs although the extent and direction of co-expression can vary between RBPs and among members of the same RBP family. They show strong association with their targets at both protein and transcript levels, however a higher level of association was observed at the protein level. Most of the RBPs show different level of association at the protein and transcript level with only 20% of them showing similar trends at both the levels. Intensity of the clip signal, number of RNA binding domains and location of the binding site on the transcript prove to be important features which can explain the association observed at the protein level while number of target transcripts, number of protein coding transcripts for the selected RBP and length of selected protein coding transcript explain the associations seen at the transcript level. On further dissecting the analysis, at the transcript centric level, we observe that the type of target transcript and the distance of the binding site from the 5′ or 3′ end of the transcript are the important factors. We also found contrasting trends i. e, same RBP can be an SC at the transcript level while being classified as an SIC RBP at the protein level - classified based on the expression associations of the RBPs with their target transcripts at the transcript and protein levels. It is interesting to note that different features were found to be significant at these two levels possibly suggesting the rational for the observed differences in directionality of the associations.

In this study, we present genome-scale evidence that majority of the RBPs are correlated in expression levels with their post-transcriptionally controlled target transcripts in both the human and yeast genomes. To our knowledge this is the first study to report such an association in post-transcriptional regulatory networks and strengthens our understanding of the relationship between RBPs and their cognate targets. Our observations suggest that in disease conditions, expression associations between RBPs and target transcripts are likely altered. Hence, prognostic RBPs can be identified by comparing the extent and number of associations in healthy versus disease expression cohorts, enabling a means of rapidly profiling for RBP biomarkers in developmental diseases, cancer and other complex disorders^4^,^5^,^33^. In conclusion, our study provides a deeper insight into the behavior of various RBPs in the context of post-transcriptional regulatory networks. Thus, providing a roadmap for the identification of different post-transcriptional regulatory patterns thereby enabling rational design of experiments pertaining to protein – RNA associations.

## Materials and Methods

### Datasets employed for human RBP binding sites as well as tissue-specific RNA and protein expression levels

To obtain a comprehensive understanding of the RBP-RNA interaction networks on a genome- wide scale and to study the characteristics of binding sites of RBPs on their target RNAs, we downloaded Crosslinking Immunoprecipitation followed by high-throughput sequencing (CLIP-Seq) data from CLIPdb database^28^ for 60 RBPs in humans. Although there is data for 63 RBPs in CLIPdb, we limited our analysis to those RBPs with high quality CLIP-seq data, limiting the number to 60 RBPs. We obtained the complete set of 217,426 annotated transcripts for the human genome from Ensembl using Biomart^50^,^51^. We mapped the binding sites onto the Ensembl HG19 version 79^51^ of the human genome to find all the target RNAs for each RBP. RNA-seq data for 16 human tissues from Illumina’s Human Body Map (HBM) 2.0^52^,^53^,^54^ was downloaded from ArrayExpress database (http://www.ebi.ac.uk/arrayexpress) under the accession number E-MTAB-513. To study the correlation of protein expression levels of RBPs with RNA levels of RBP target transcripts, we downloaded mass spectrometry based proteomic data for over 17,000 human proteins across 30 tissues/cell lines (17 adult, 7 fetal tissues and 6 hematopoietic cells) from Human Proteome Atlas (HPA)^55^.

### Constructing RBP-RNA regulatory network

Several studies have shown that RBPs bind 200–300 nucleotides around the observed splice sites, which generally possess the identifiable sequence features^56^,^57^. We therefore considered a transcript to be a target of a RBP, if and only if the binding sites of the RBP fall within the 300 bps flanking regions or 300 bps downstream regions of at least one of its annotated exonic start or end co-ordinates. Based on this criterion, if at least one exon is mapped with a RBP’s binding site, the corresponding transcript is considered its target transcript. This allowed us to build a RBP – target transcript network for each RBP which was used in the downstream analysis (see Fig. 1). The union of unique number of transcripts targeted by each of the 60 RBPs is 121,131 transcripts. The number of target transcripts for each RBP based on the built regulatory network is listed in Table 1.

### Correlation analysis to study the association between each RBP and its target transcripts

Transcript level expression was quantified for the downloaded RNA-seq data using Sailfish v0.6.3^58^. TPM (transcripts per million) values were considered for the quantification of expression levels of all the Ensembl annotated transcripts in the human genome. The target transcripts which were mapped as described above for each of the 60 RBPs, were then matched with the genome-wide transcript levels across tissues and were divided into 3 categories for each RBP – 1) RBP target transcripts 2) RBP control matched – defined as the set of randomly selected transcripts in the expression compendium equal in number as the number of target transcripts and 3) RBP control all – defined as the set of all the transcripts which were not annotated to be targeted by a RBP based on CLIP-seq data.

To identify a representative protein coding transcript encoding for an RBP among all the annotated transcripts in the RNA-seq data, a protein coding transcript with the highest mean expression level across all the 16 tissues was chosen. Spearman correlation was calculated between the transcript level expression of each RBP and every target as well as non-target transcript’s expression levels across the 16 tissues to generate correlation coefficients for the three different categories of transcripts namely RBP target transcripts, RBP control matched and RBP control all, defined above. Similarly, for the protein expression data upon mapping the tissues from this dataset with the tissues available in the RNA-seq dataset, we found 9 common tissues and hence spearman correlations were computed between the protein expression level of each RBP and its target as well as non-target transcript’s expression level from RNA-seq data which resulted in three categories of correlation coefficients for each RBP (see Fig. 1). Boxplots were plotted to represent the differences in the extents of correlation among the three categories of transcripts for each RBP and corresponding pairwise Wilcoxon test p-values computed using R to understand the significance of the observed patterns.

### Identification of factors contributing to the observed association between RBPs and their target transcripts

To understand what factors and the extent to which they might be contributing to the observed correlation patterns at the transcript and protein levels of RBPs, we employed multivariate modelling at two different levels, referred to as the RBP centric level and the transcript centric level in this study. We employed three different feature selection/reduction methods to identify the robust set of contributing features, namely – the lm function, the step LR function and the ElasticNet^59^ package in R. The lm function is an inbuilt function in R with a typical model in the form response ~ terms and is used to fit linear models and carry out regression and analysis of covariance and variance. Contrary to the lm method where all the features are included in the analysis, step function is an automated procedure where at the end of each step, variables with the most insignificant p – values are dropped and the procedure stops when the remaining features have a p – value significantly defined by a threshold value alpha. Ridge regression (L2 regularization term)^60^ uses all input features to fit a model, while LASSO (L1 regularization term)^61^ tries to find the most optimal fit. Elastic Net is a regularized regression modelling method which combines the above two methods and optimizes the bias and variance discrepancies between lasso and ridge.

At the RBP centric level, our goal was to understand and identify the general features which can likely explain the observed association between RBPs and their targets. These included nine features namely number of target transcripts controlled by an RBP, median CLIP signal of a RBP, number of RNA-binding domains in a RBP, number of documented protein interactions of a RBP, number of protein coding transcripts encoded by a RBP, total number of annotated transcripts by the gene encoding for RBP, length of the selected protein coding transcript, median of all the distances between the binding site of RBP and to the closest end of the transcript and correlation between mRNA-protein levels of an RBP, which could play a role in influencing the median correlation coefficient of all the target transcripts for each RBP. At the transcript centric level, our goal was to uncover the contribution of the transcript specific features such as CLIP signal on the target transcript, distance of binding site with respect to the 5′ or 3′ end of the transcript, length as well as type of the transcript on the correlation coefficient of each target transcript for each RBP. A detailed description of each of these features is listed in Tables 2 and 3 for RBP centric and transcript centric models respectively. RNA-binding domain annotations for RBPs were obtained from a previous study^5^ and the number of protein – protein interactions for each RBP was calculated by constructing a protein – protein interaction network using interaction data from the BIOGRID database^48^.

### Post-transcriptional regulatory network of RBP – RNA interactions in yeast and analysis of correlation patterns

We hypothesized that the observed correlation patterns of RBPs with their target transcripts is conserved across species. To test this hypothesis, we used the post-transcriptional regulatory network of 69 RBPs and 24,932 RBP-RNA interactions in the yeast genome^43^. We downloaded RNA-seq data from a previous study generated under 18 different environmental conditions in yeast, with each condition having two biological replicates^62^. In particular, the data available at http://downloads.yeastgenome.org/published_datasets/Waern_2013_PMID_23390610/ for *S. cerevisiae* strain S288C reference genome sequence version R64-1-1^63^ were downloaded from Saccharomyces Genome Database^64^. Raw data was quality filtered, aligned using Tophat^65^ and expression levels of transcripts quantified using Cufflinks^66^. Similar analysis as implemented for the human genome was executed to identify the association between RBPs and their target/non-target RNAs. Boxplots were plotted using R. We limited our analysis to only 65 RBPs as opposed to 69 RBPs since for four of the yeast RBPs, only 1 target was detected with negligible expression levels.

## Additional Information

**How to cite this article**: Nishtala, S. *et al.* Dissecting the expression relationships between RNA-binding proteins and their cognate targets in eukaryotic post-transcriptional regulatory networks. *Sci. Rep.* **6**, 25711; doi: 10.1038/srep25711 (2016).

## Supplementary Material

Supplementary Information

## Figures and Tables

**Table 1:** List of human RBPs, their number of target transcripts and source of CLIP data.

RBP	# targets	Source of CLIP-Seq data (References)
AGO1	17,206	67,68
AGO2	64,425	67, 68, 69, 70, 71, 72, 73, 74, 75, 76
AGO3	5,849	67
AGO4	1,517	67
ALKBH5	1,685	77
ATXN2	6,715	78
C17ORF85	2,199	77
CAPRIN1	7,506	77
CPSF1	9,132	79
CPSF2	1,813	79
CPSF3	2,832	79
CPSF4	3,486	79
CPSF6	38,973	79
CPSF7	45,556	79
CSTF2	36,829	79,80
CSTF2T	32,893	79
DGCR8	14,587	81
EIF4A3	35,729	82
ELAVL1	55,432	69,83, 84, 85
EWSR1	6,702	86,87
EZH2	116	88
FBL	4,019	89
FIP1L1	34,643	79
FMR1	10,732	90
FUS	2,150	87,91,92
FXR1	2,940	90
FXR2	9,230	90
HNRNPA1	9,587	93
HNRNPA2B1	2,013	93
HNRNPC	18,806	94,95
HNRNPD	6,121	96
HNRNPF	3,461	93
HNRNPH	3,608	97
HNRNPM	3,051	93
HNRNPU	10,318	93,98
IGF2BP1	15,328	67
IGF2BP2	10,668	67
IGF2BP3	10,022	67
LIN28A	16,860	42,99
LIN28B	21,277	99,100
MOV10	7,523	101
NOP56	2,007	89
NOP58	7,879	89
NUDT21	40,541	79
PTBP1	25,385	102,103
PTBP2	16,956	102,103
PUM2	1,938	67
QKI	1,358	67
RTCB	6,376	77
SRRM4	10,026	103
TAF15	3,197	87,104
TARDBP	12,138	105
TIA1	11,977	106
TIAL1	23,854	106
TNRC6A	828	67
TNRC6B	643	67
TNRC6C	859	67
WDR33	1,626	107
YTHDF2	15,474	108
ZC3H7B	13,294	77

Table shows a list of all the 60 RBPs employed in the analysis, the number of transcripts targeted by each of them and references to the studies which provide the CLIP-Seq data^28^. This list was generated by mapping the binding sites of each RBP with exonic coordinates and obtaining the corresponding transcripts of the mapped exons.

**Table 2:** Different features employed to study their contribution towards observed correlation between RBPs and their post-transcriptional targets, in the RBP centric modelling.

Variable	Feature name	Description
Response	Median correlation coefficient of target transcripts	This was calculated by taking the median of all the correlation coefficients of each RBP with its target transcripts.
Predictor	Number of target transcripts	The number of target transcripts of each RBP was obtained by the mapping the co-ordinates of the binding sites onto the annotated transcripts as described in materials and methods.
	Median CLIP Signal	CLIP peaks in the bed format obtained from CLIPdb^28^ come with a P- value signifying the intensity of the CLIP binding for each binding site. The median P - value of the binding sites which were mapped to the targets, for each RBP, was calculated.
	Number of RNA binding domains	Number of RNA binding domains for each RBP was obtained from a previous study describing the compendium of human RBPs 5.
	Number of protein-protein interactions	Human protein - protein interaction network was constructed using data from BIOGRID^48^. Then, for each RBP, its number of interacting partners was computed.
	Number of protein coding transcripts	The number of protein coding transcripts documented for each RBP was obtained from Ensembl^51^.
	Number of annotated transcripts	Total number of transcripts (protein coding, processed transcript, etc.,) documented for each RBP was also obtained from Ensembl^51^.
	Length of the selected protein coding transcript	The length of the selected protein coding transcript used for computing correlation of each RBP with target transcripts and control transcripts was also obtained from Ensembl^51^.
	Median distance of binding site from transcript	The closest distance of the start of the binding site from either ends on the transcript was calculated for each target transcript. The median value of this distance was then calculated for each RBP.
	mRNA - protein correlation	The protein expression of each RBP was correlated with the mRNA expression across nine tissues with both RNA and protein expression data.

For each feature details about how it was computed is also listed.

**Table 3:** Different features employed to study their contribution towards observed correlation between RBPs and their post-transcriptional targets, in the transcript centric modelling.

Variable	Feature name	Description
Response	Correlation coefficient of RBP with target transcript	For each RBP, the correlation coefficient of the selected protein coding transcript (or protein expression at protein level) with each target transcript was selected as the response variable.
Predictor	CLIP Signal	CLIP peaks in the bed format obtained from CLIPdb^28^ come with a P- value signifying the intensity of the CLIP binding for each binding site. The median P - value of the binding sites which were mapped on to the target, for each RBP, was calculated.
	Distance of binding site from 5′ end	The distance of the start of the binding site from the 5′ end of each target transcript was calculated.
	Distance of binding site from 3′ end	The distance of the start of the binding site from the 3′ end of each target transcript was calculated.
	Transcript Length	The length of each target transcript was obtained from Ensembl^51^.
	Transcript Type	The biotype of each target transcript was also obtained from Ensembl^51^.

For each feature details about how it was computed is also listed.
