# Supplementary material for: Dissecting the expression relationships between RNA-binding proteins and their cognate targets in eukaryotic post-transcriptional regulatory networks
Source: Sci Rep. 2016 May 10;6:25711. doi: 10.1038/srep25711 (PMC4861959; doi:10.1038/srep25711)
Supplement: Supplementary Information [file srep25711-s1.pdf]

Dissecting the expression relationships between RNA-binding proteins and their cognate targets in eukaryotic post-transcriptional regulatory networks

Sneha Nishtala[1], Yaseswini Neelamraju[1], Sarath Chandra Janga[1,2,3,*]

[1]Department of Bio Health Informatics, School of Informatics and Computing, Indiana University Purdue University, 719 Indiana Ave Ste 319, Walker Plaza Building, Indianapolis, Indiana 46202

[2]Centre for Computational Biology and Bioinformatics, Indiana University School of Medicine, 5021 Health Information and Translational Sciences (HITS), 410 West 10th Street, Indianapolis, Indiana, 46202

[3]Department of Medical and Molecular Genetics, Indiana University School of Medicine, Medical Research and Library Building, 975 West Walnut Street, Indianapolis, Indiana, 46202

*_Correspondence should be addressed to: Sarath Chandra Janga (scjanga@iupui.edu)_

719 Indiana Avenue Ste 319, Walker Plaza Building

Indianapolis, Indiana - 46202

Tel: +1-317-278-4147, Fax: +1-317-278-9201

Legends:

Supplementary figure 1: Figure showing correlation patterns for all the 60 human RBPs organized into SC, SIC and NSC categories when the transcript levels of RBPs and their target/non-target transcripts are considered. RNA-sequencing based expression data from the Human Body Map (HBM) project for16 human tissues was employed in this analysis.

Supplementary figure 2: Figure showing correlation patterns of all the 65 yeast RBPs organized into SC, SIC and NSC categories using the RNA-seq based expression profiles across 18 conditions and a constructed post-transcriptional regulatory network for yeast RBPs.

Supplementary figure 3: Figure showing correlation patterns of all the 58 RBPs organized into SC, SIC and NSC categories using the protein expression levels of RBPs in matched tissues with RNA-seq data.

Supplementary figure 4: Standardized coefficients from the feature selection analysis using elastic net for RBP centric modelling. a) At the transcript level, nTT: number of target transcripts, nPCT: number of protein coding transcripts, LPCT: Length of selected protein coding transcript, MPC: mRNA-protein correlation, mCS: median CLIP signal, mD: median distance of binding site from target transcript are the features in the order of importance. b) At the protein level, mCS, median CLIP signal, RBD: number of RNA binding domains, mD: median distance of binding site from transcript, LPCT: length of protein coding transcript, MPC: mRNA – protein correlation are the features in the order of importance.

Supplementary figure 5: Heatmaps showing the transcript centric modelling without clustering when a) transcript levels of RBPs are considered and b) protein levels of RBPs are considered.

Supplementary table 1: Table showing network centality properties of RBPs belonging to sync and non-sync groups using Biogrid and String database.
Sync RBPs (12) - RBPs which shows similar level of association at both the transcript and protein level belong to this group. Non-sync RBPs (31) - RBPs which are SC at transcript and SIC at protein level or vice-versa belong to this group.

Supplementary figure 1
Transcript level correlation

Significantly Congruent

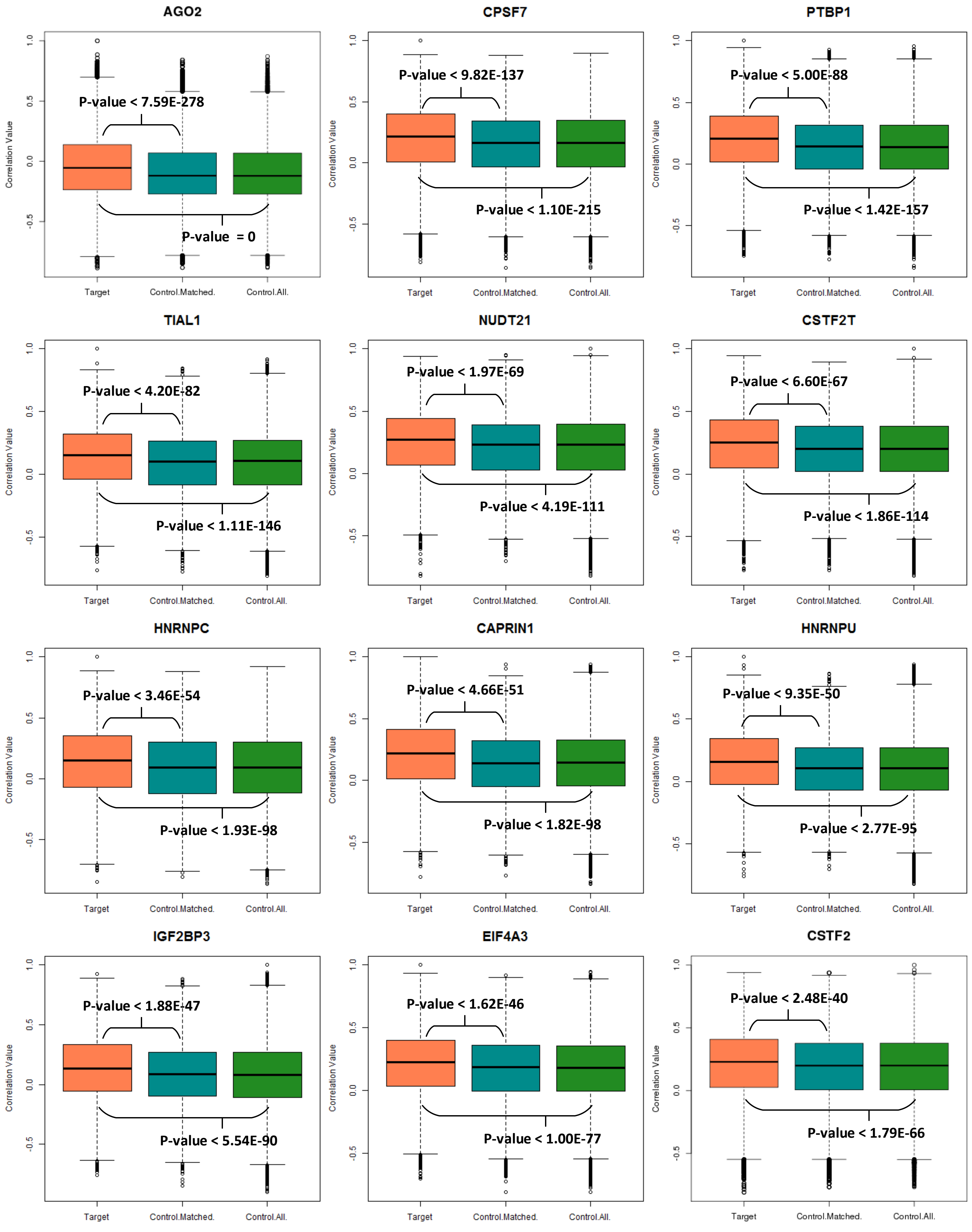

Significantly Congruent

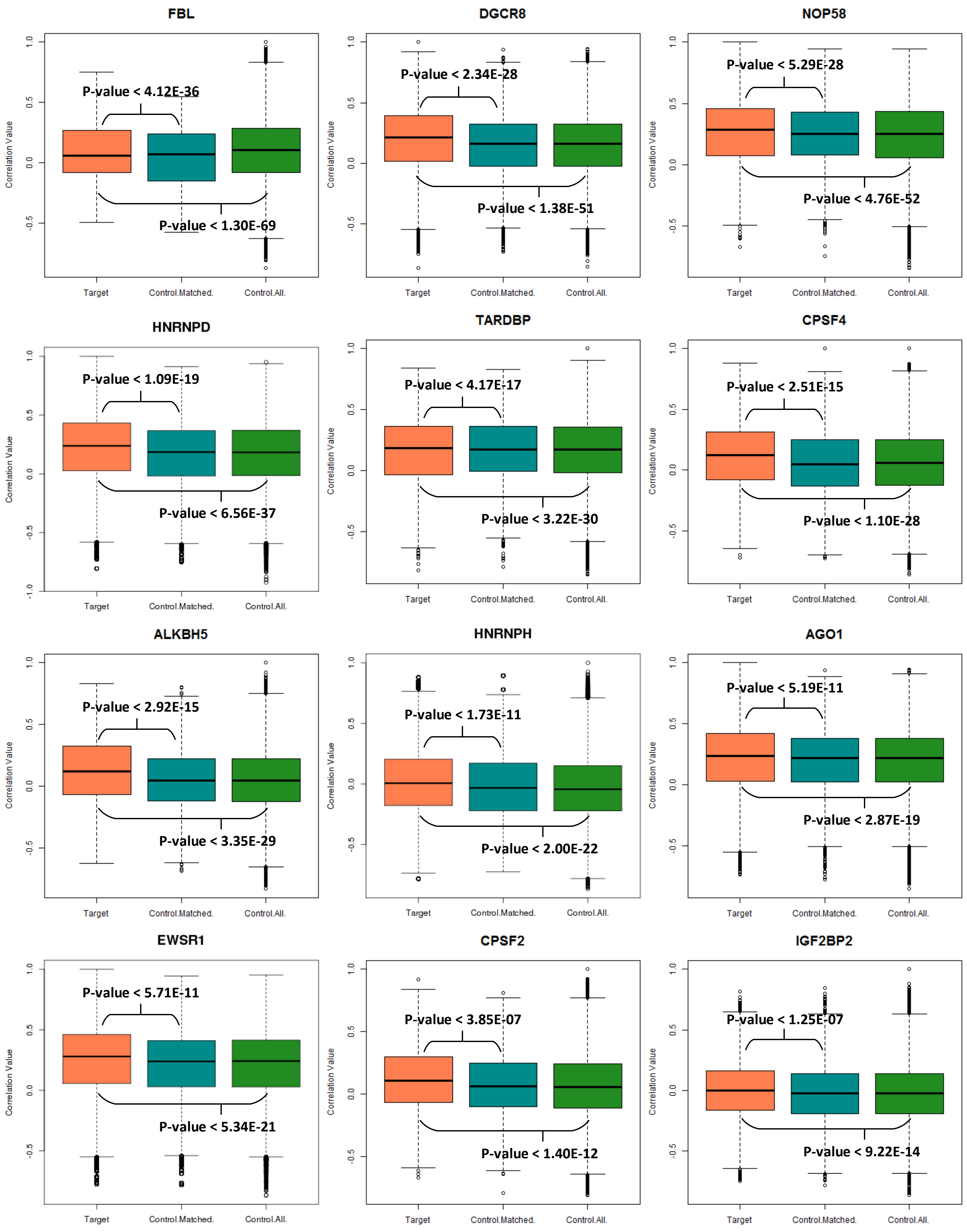

Significantly Congruent

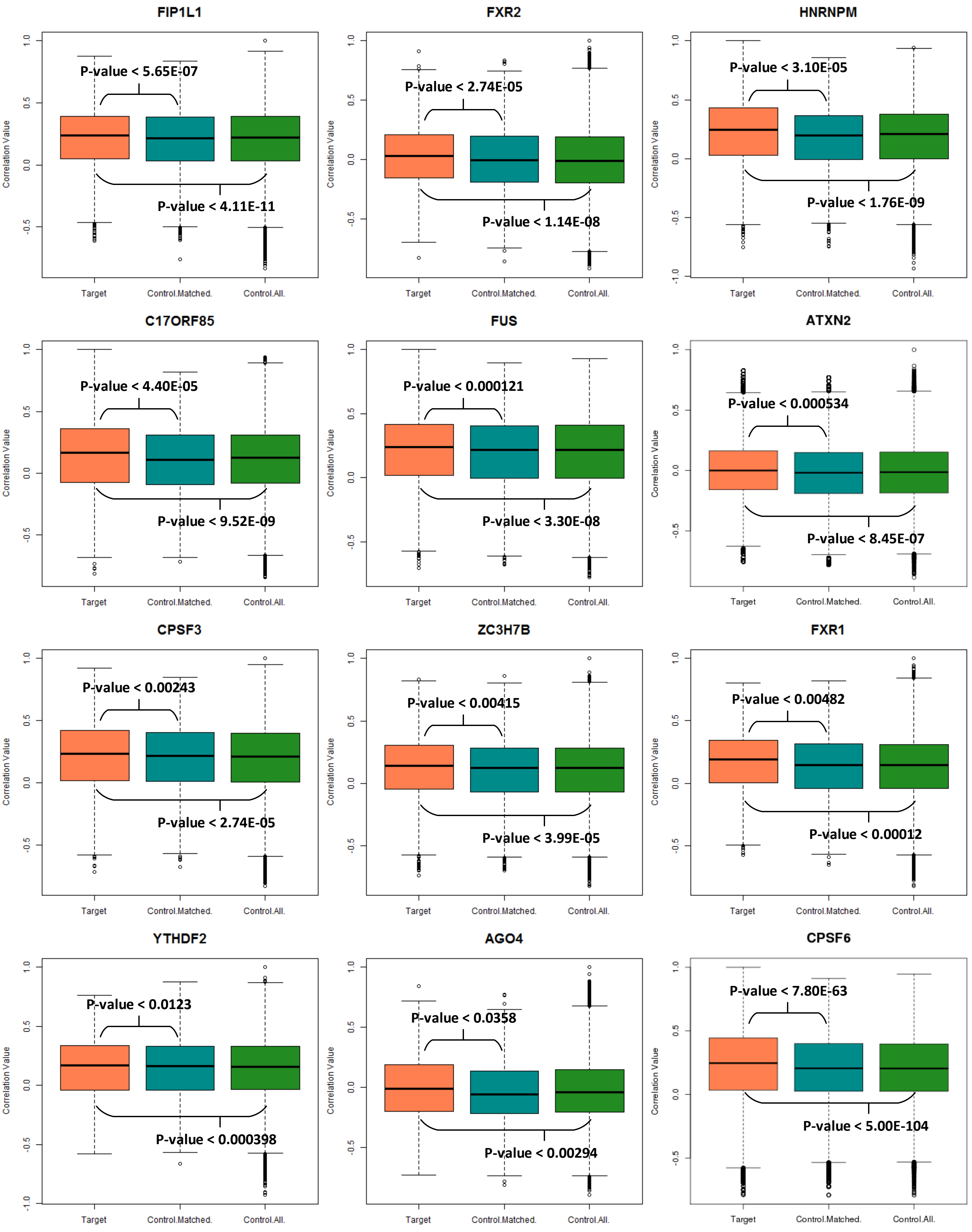

Significantly Incongruent

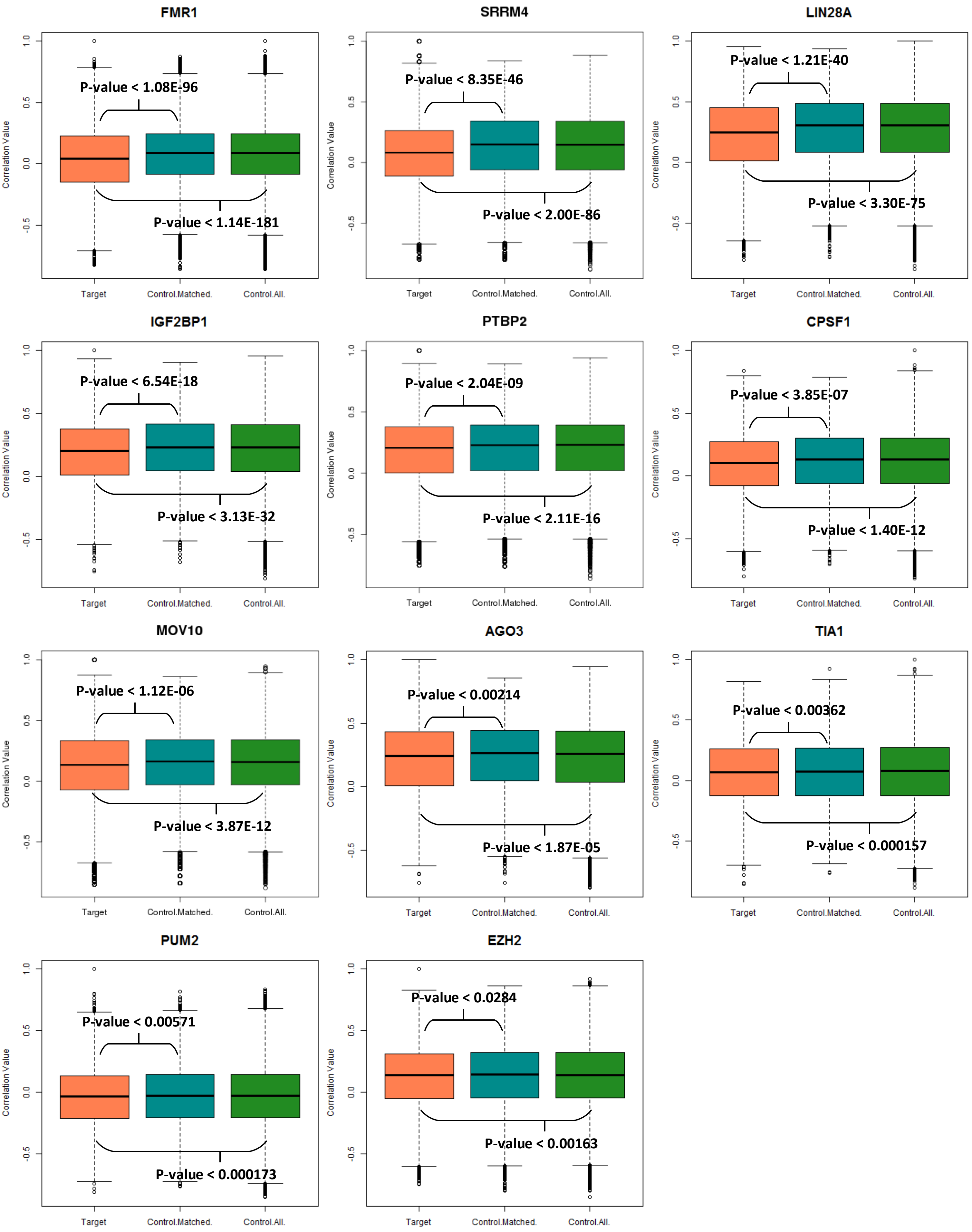

No Significant Change

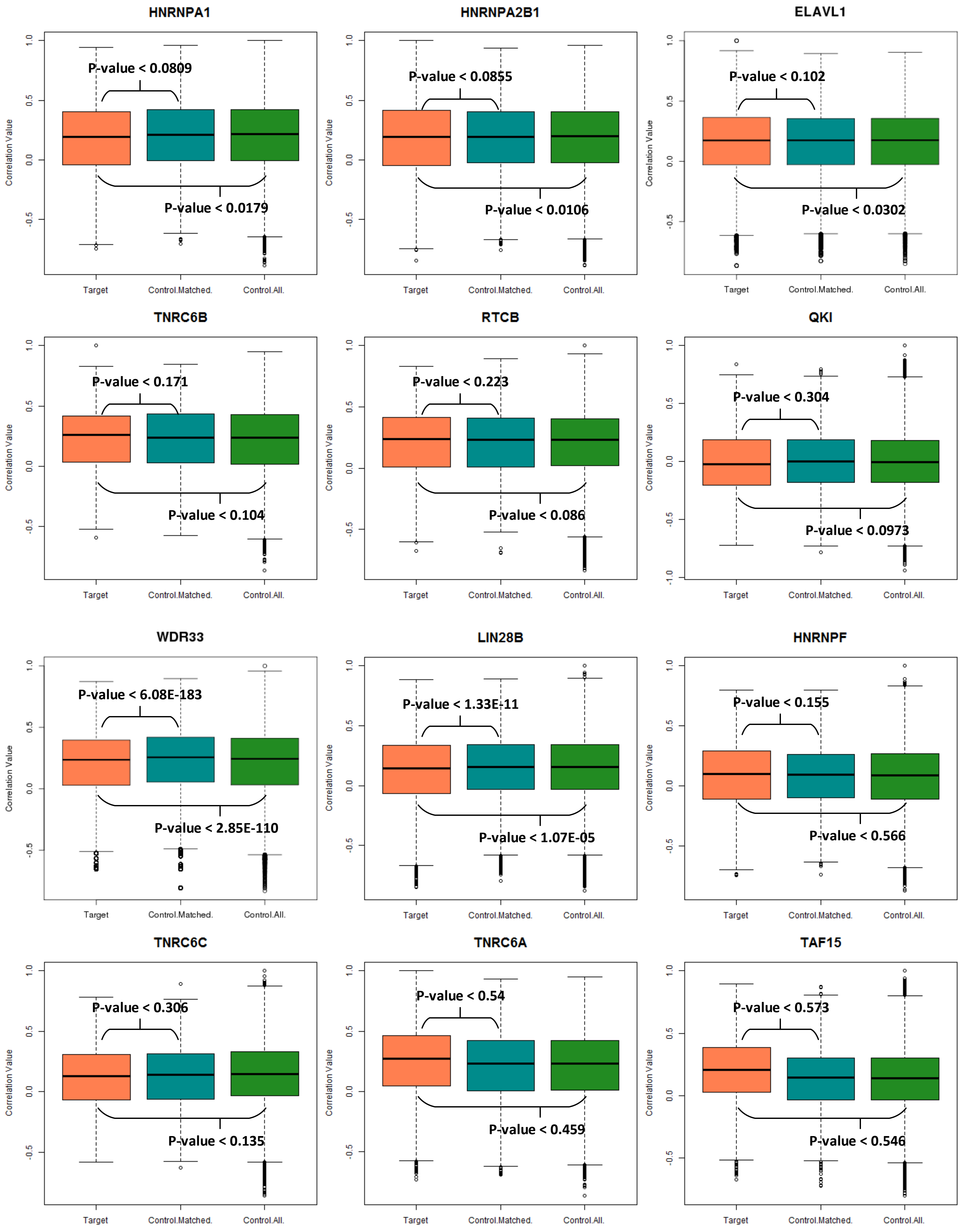

No Significant Change

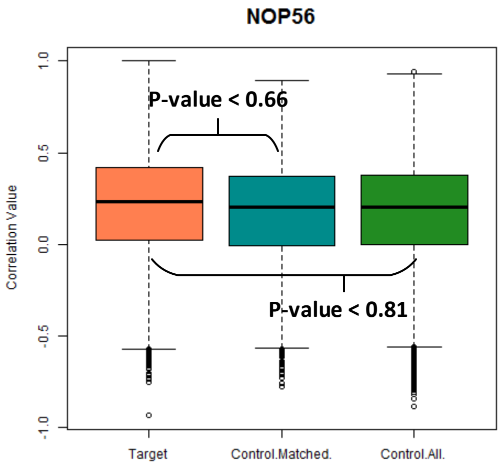

Supplementary figure 2
Yeast
Transcript level correlation

Significantly Congruent

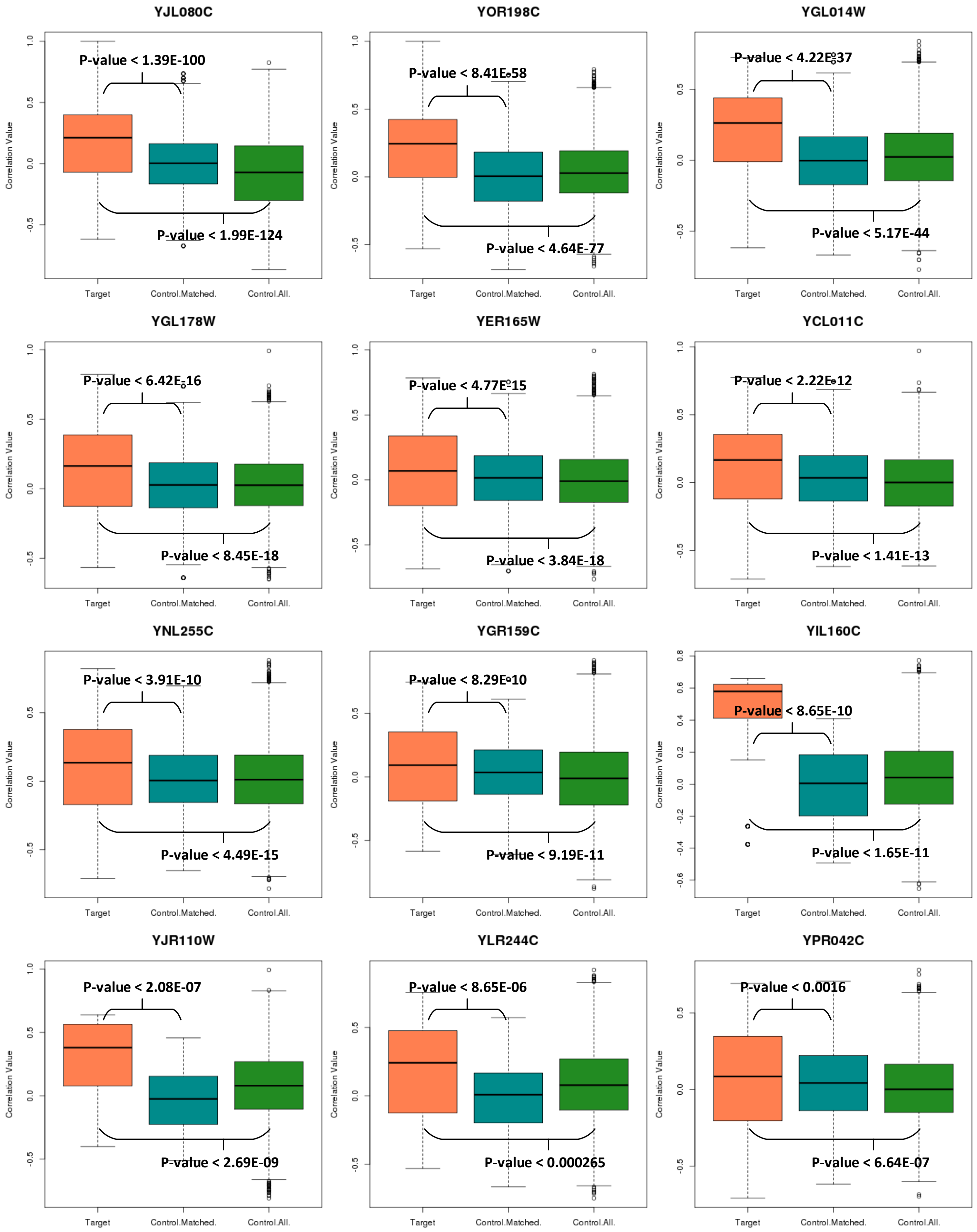

Significantly Congruent

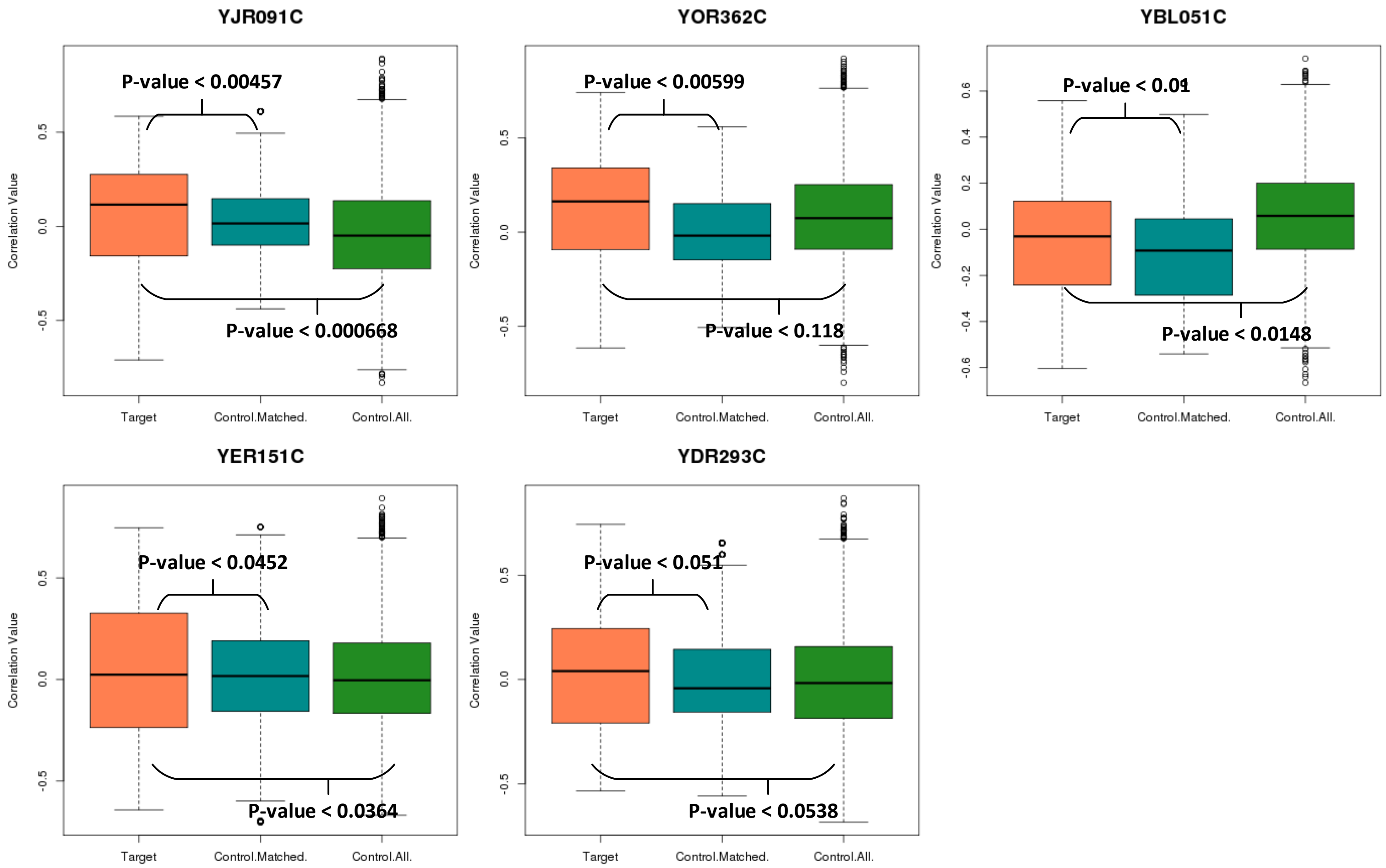

Significantly Incongruent

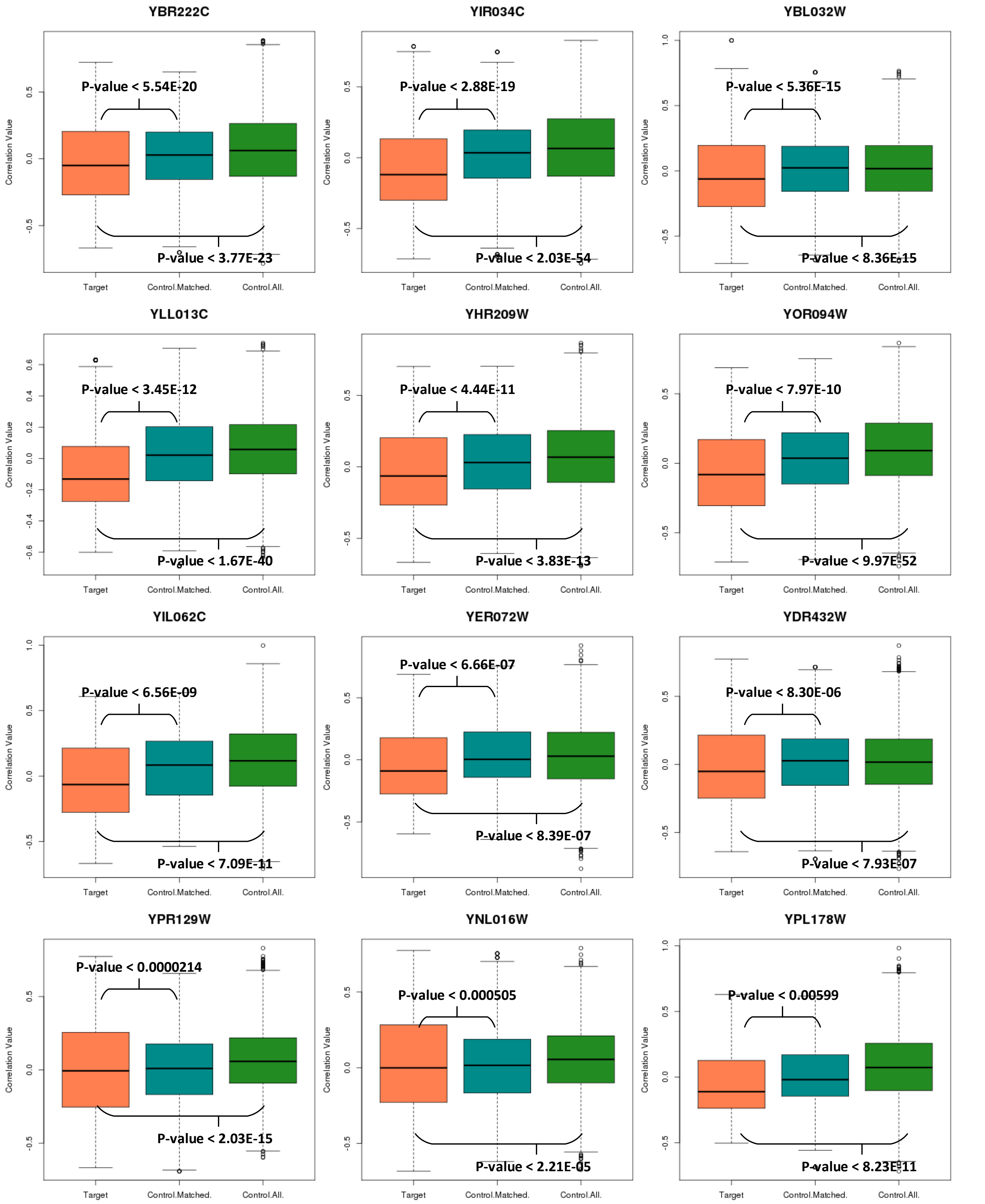

Significantly Incongruent

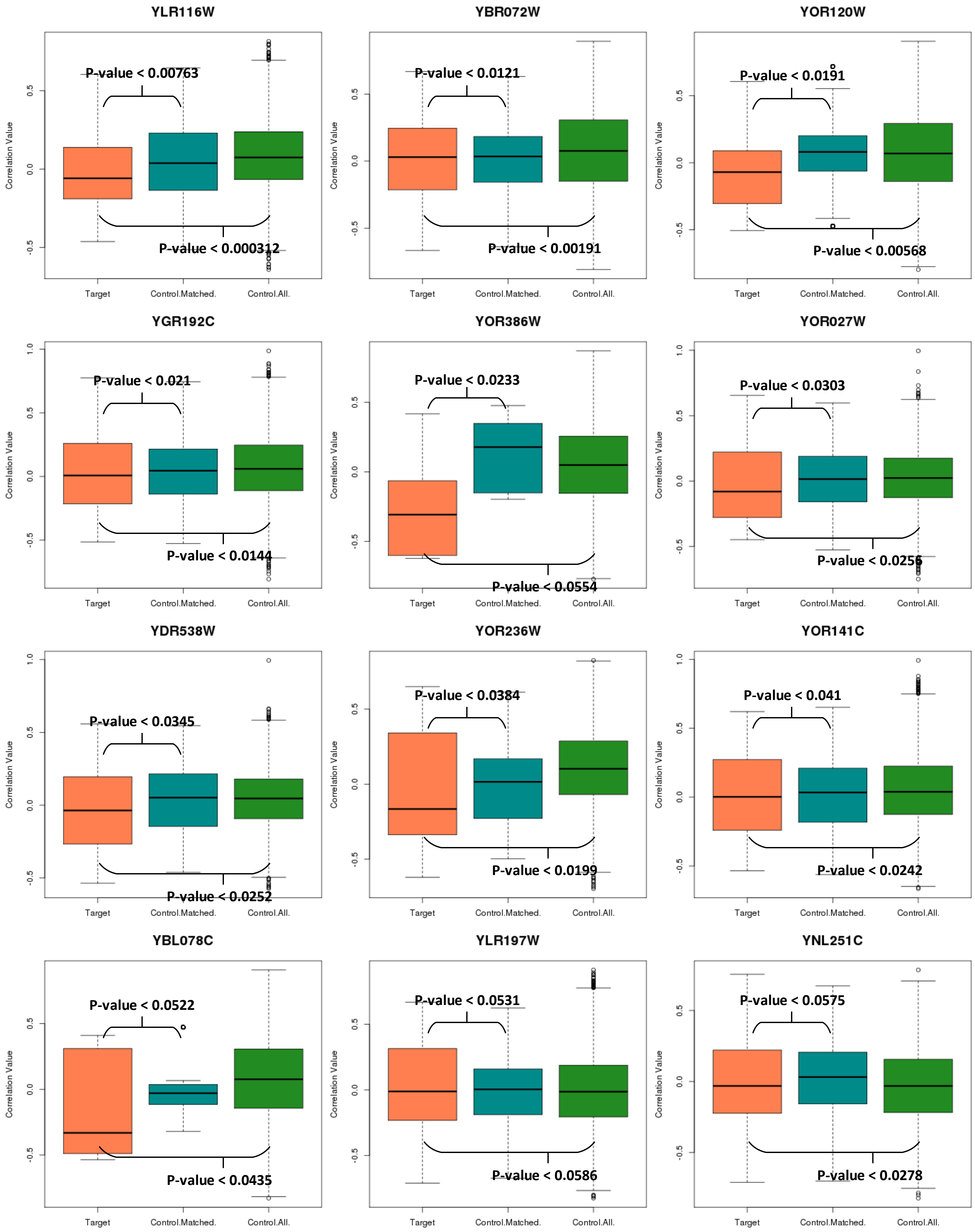

No Significant Change

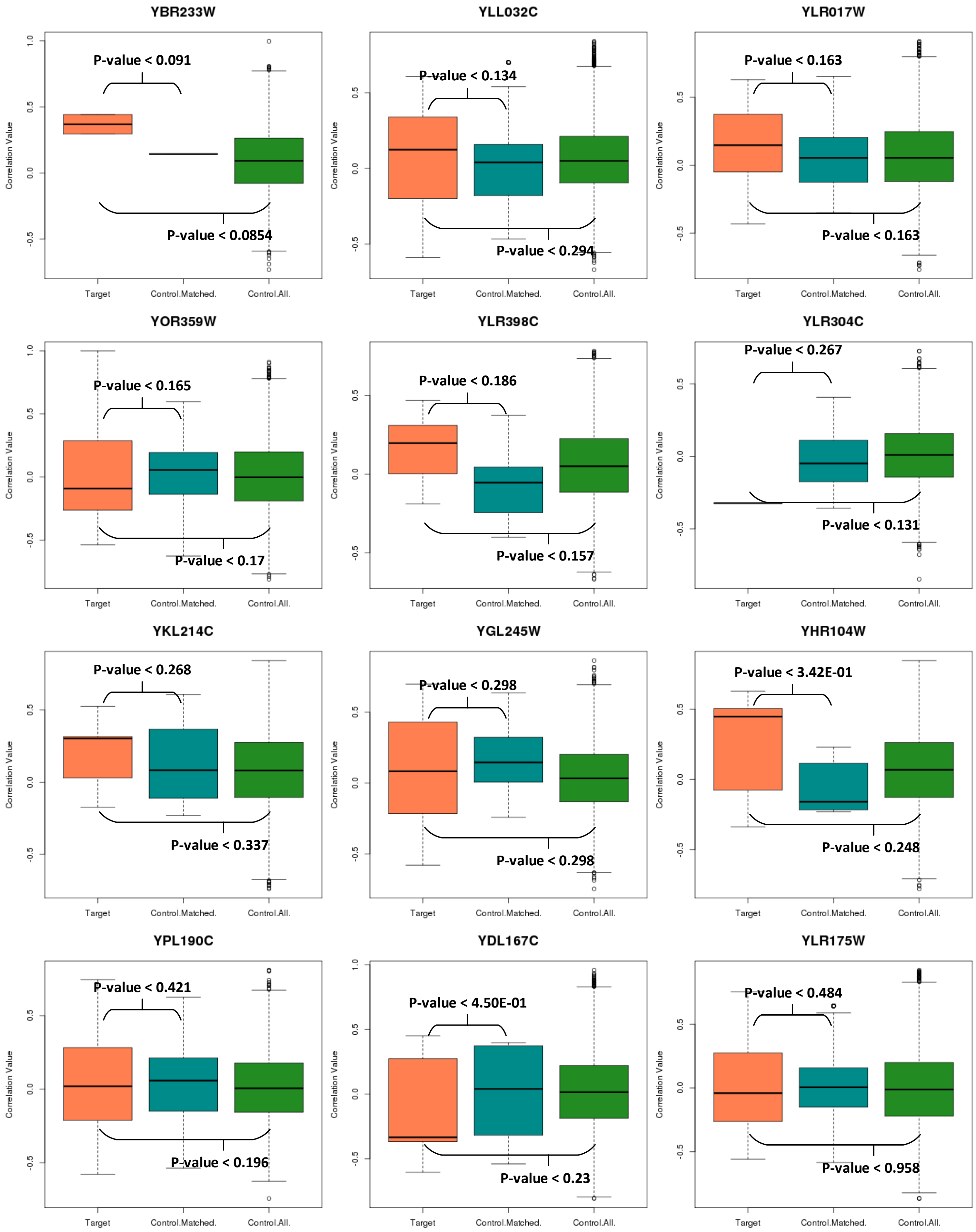

No Significant Change

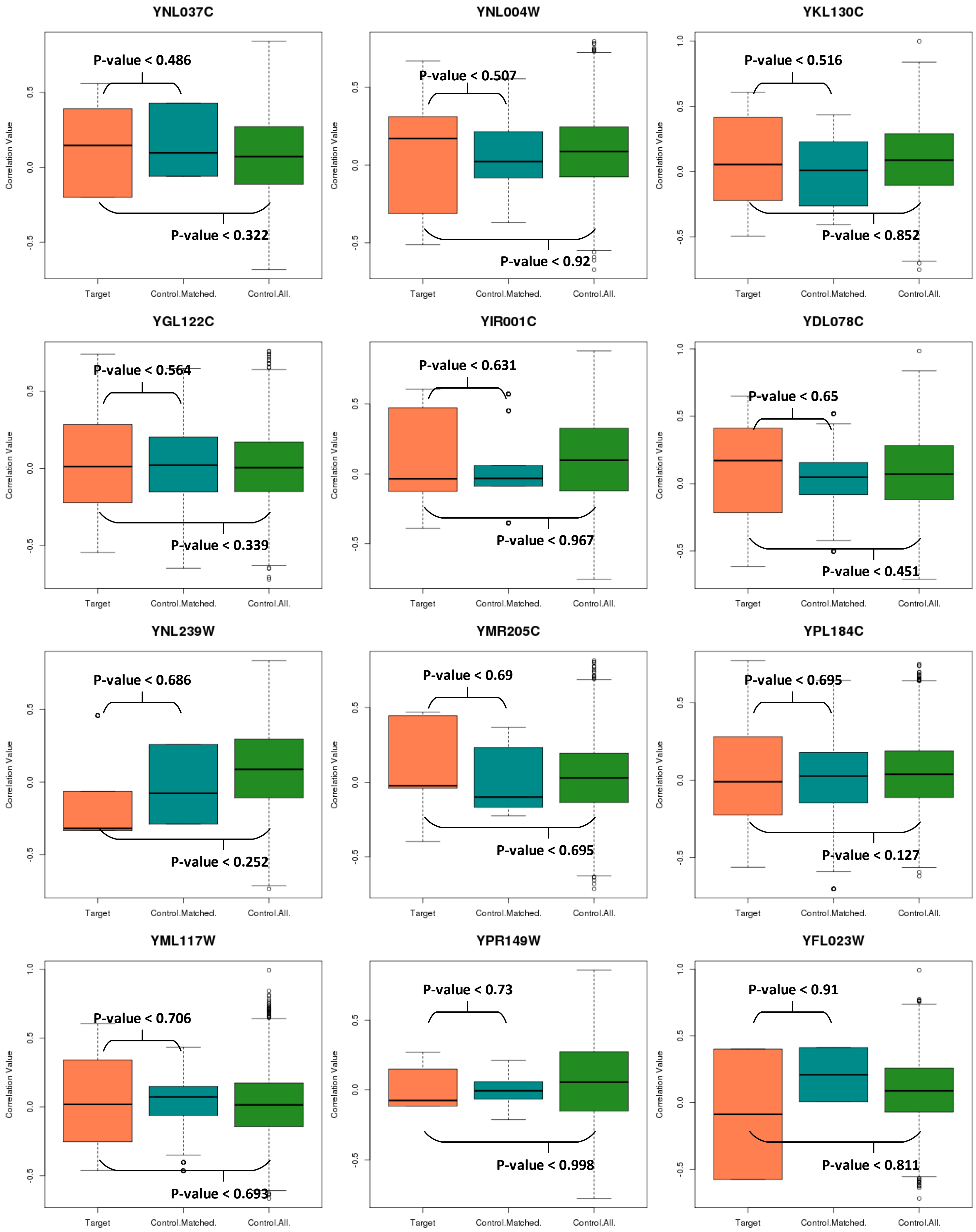

Supplementary figure 3
Protein level correlation

Significantly Congruent

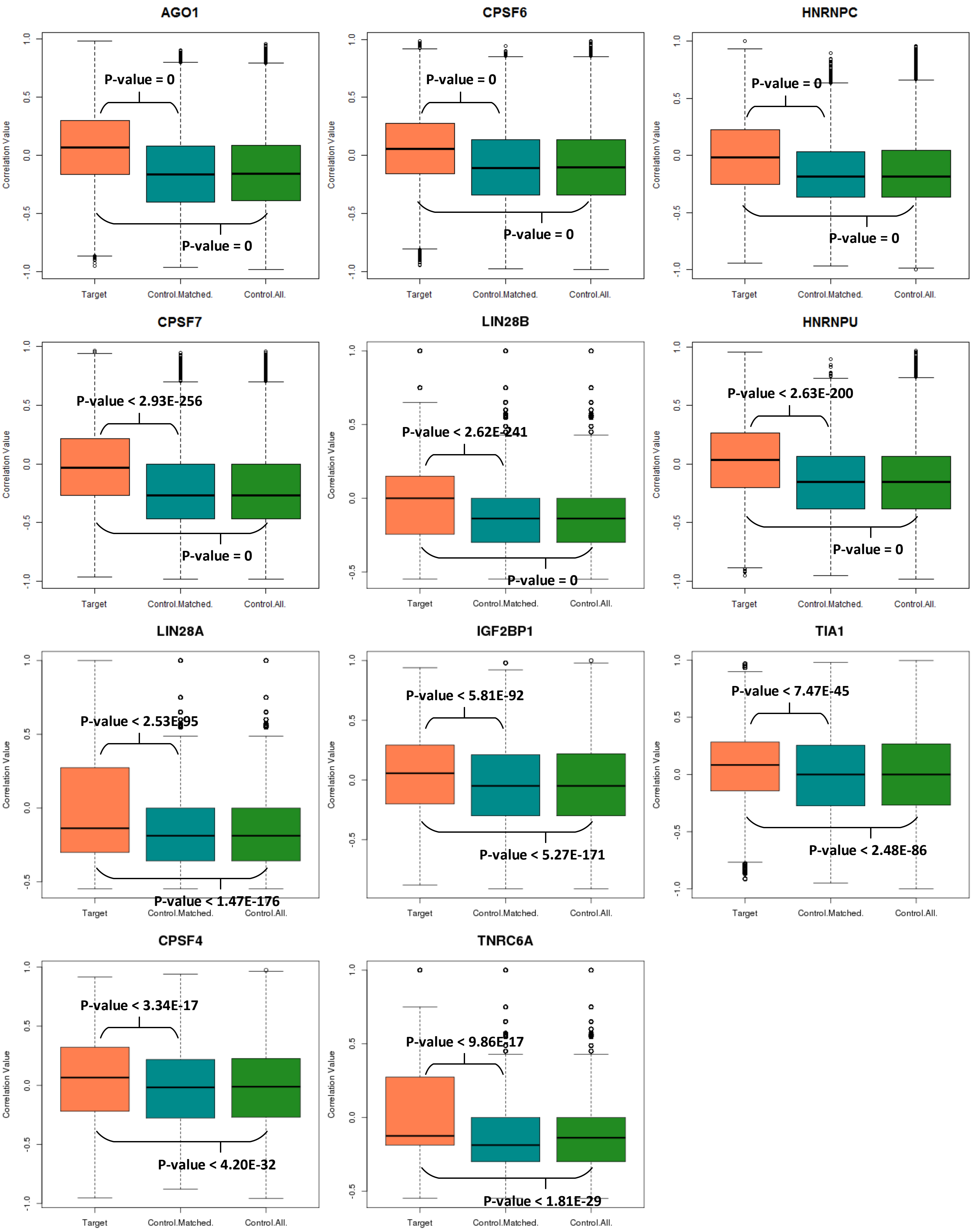

Significantly Incongruent

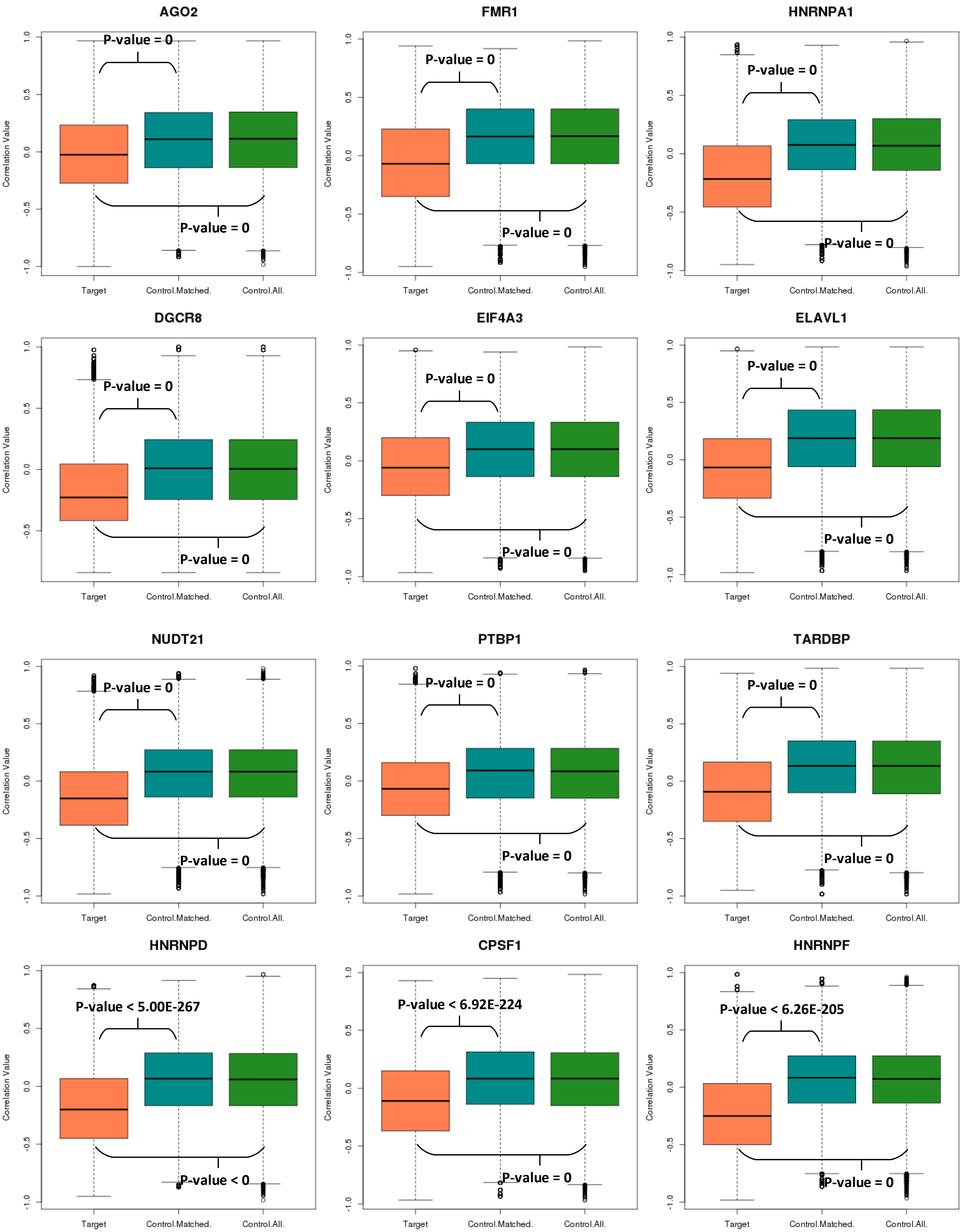

Significantly Incongruent

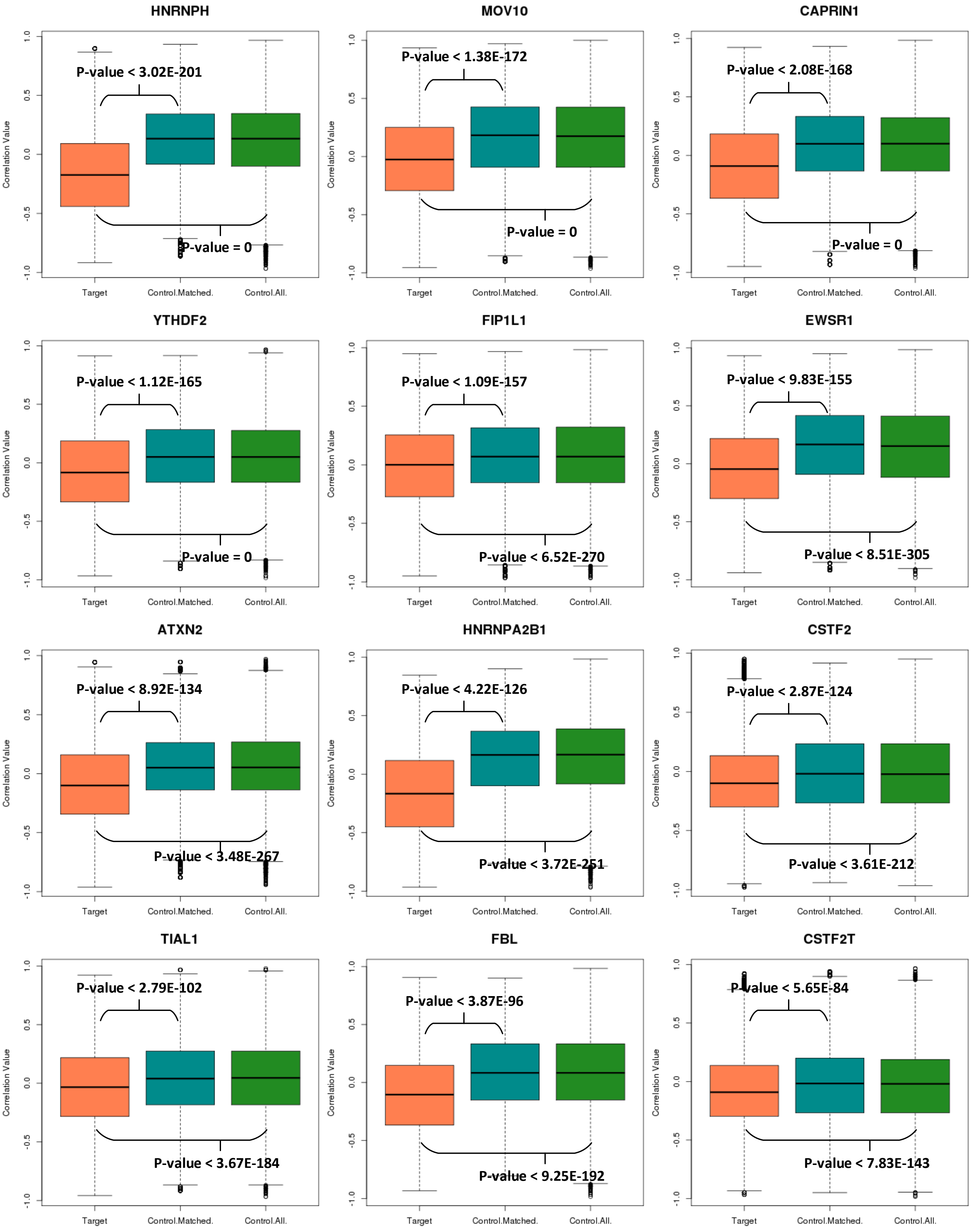

Significantly Incongruent

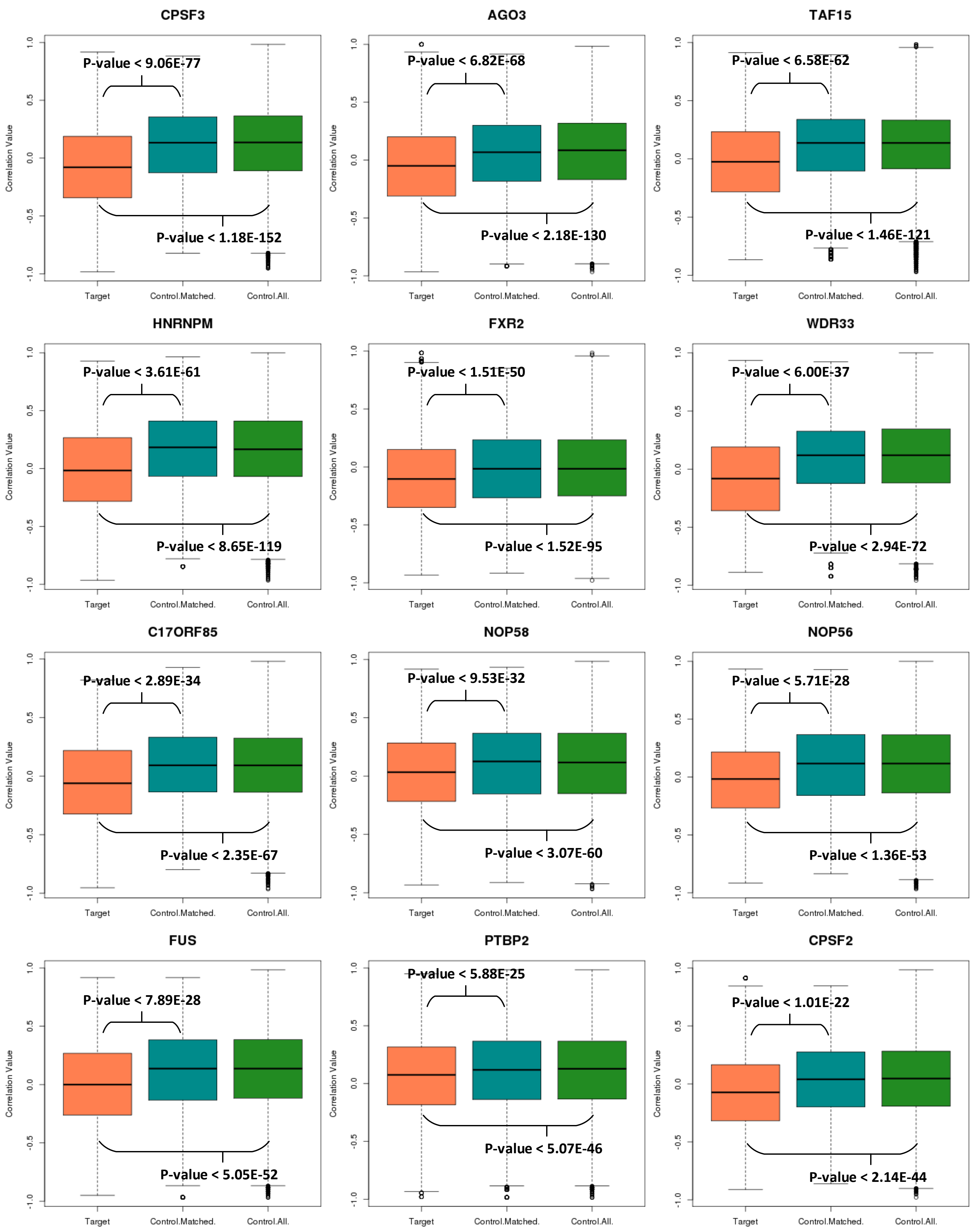

Significantly Incongruent

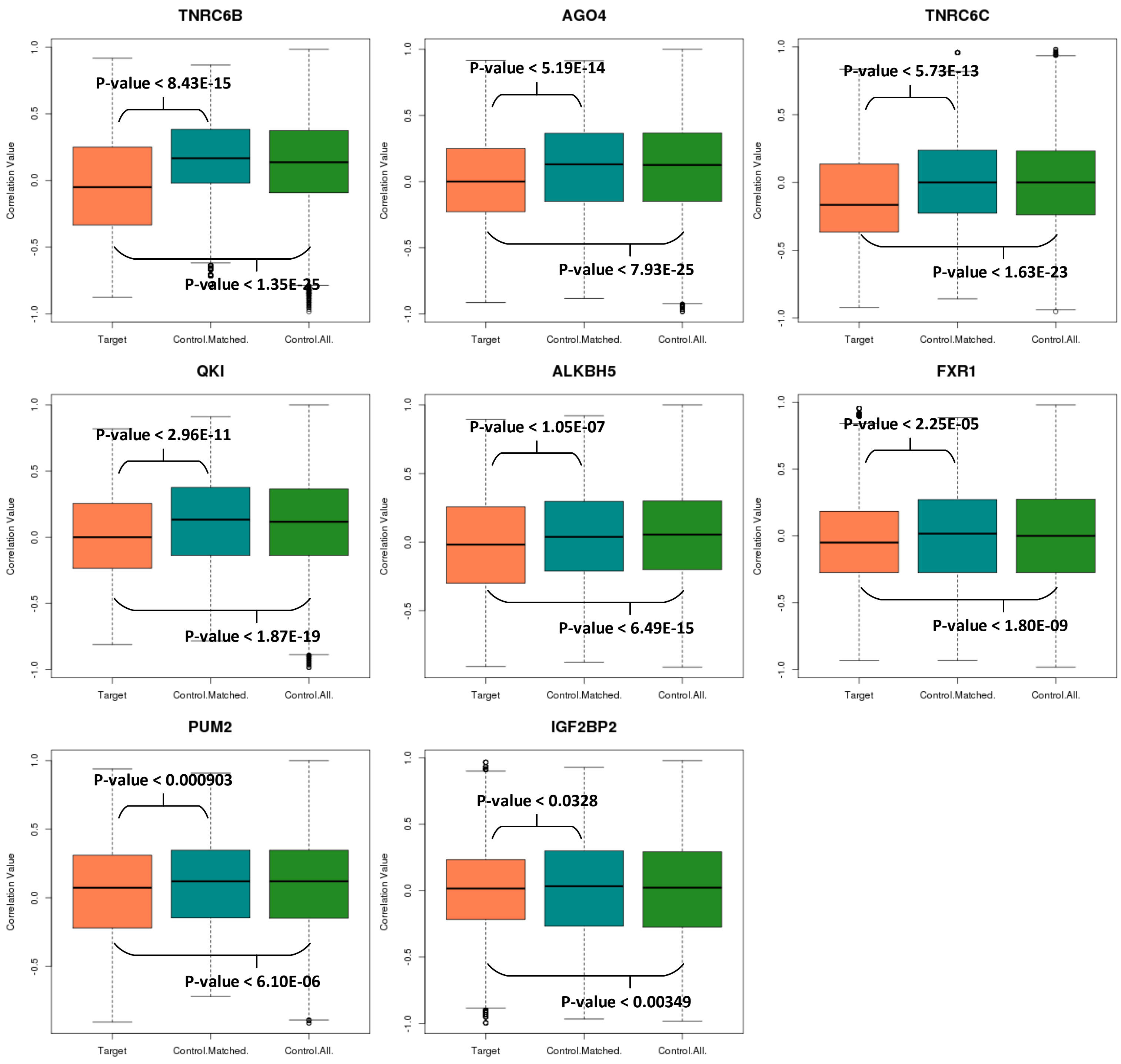

No Significant Change

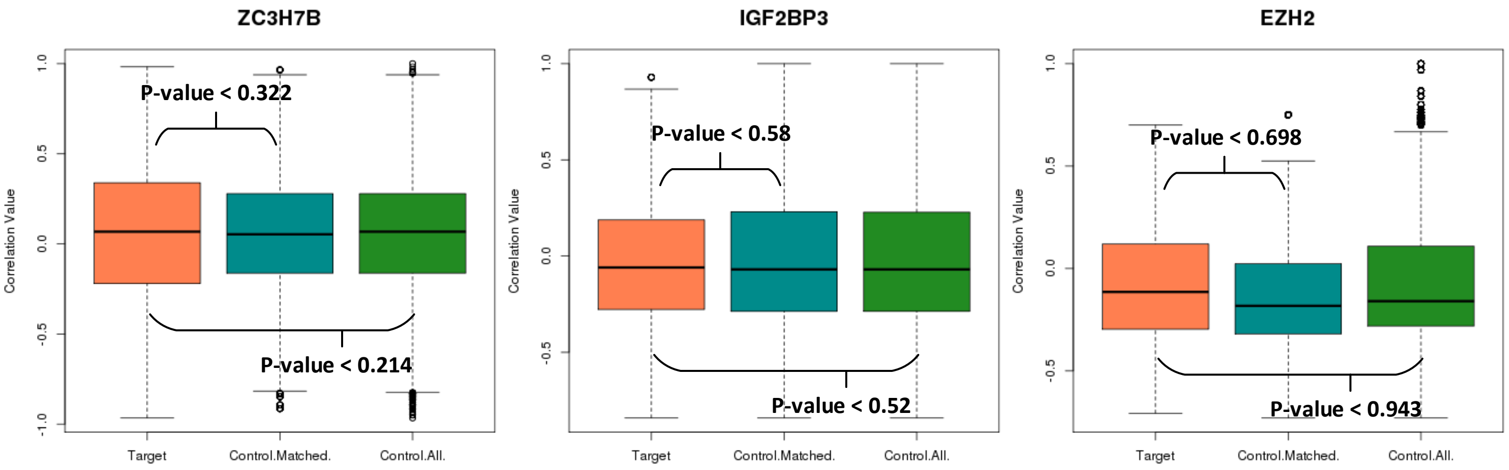

Supplementary figure 4:

a)

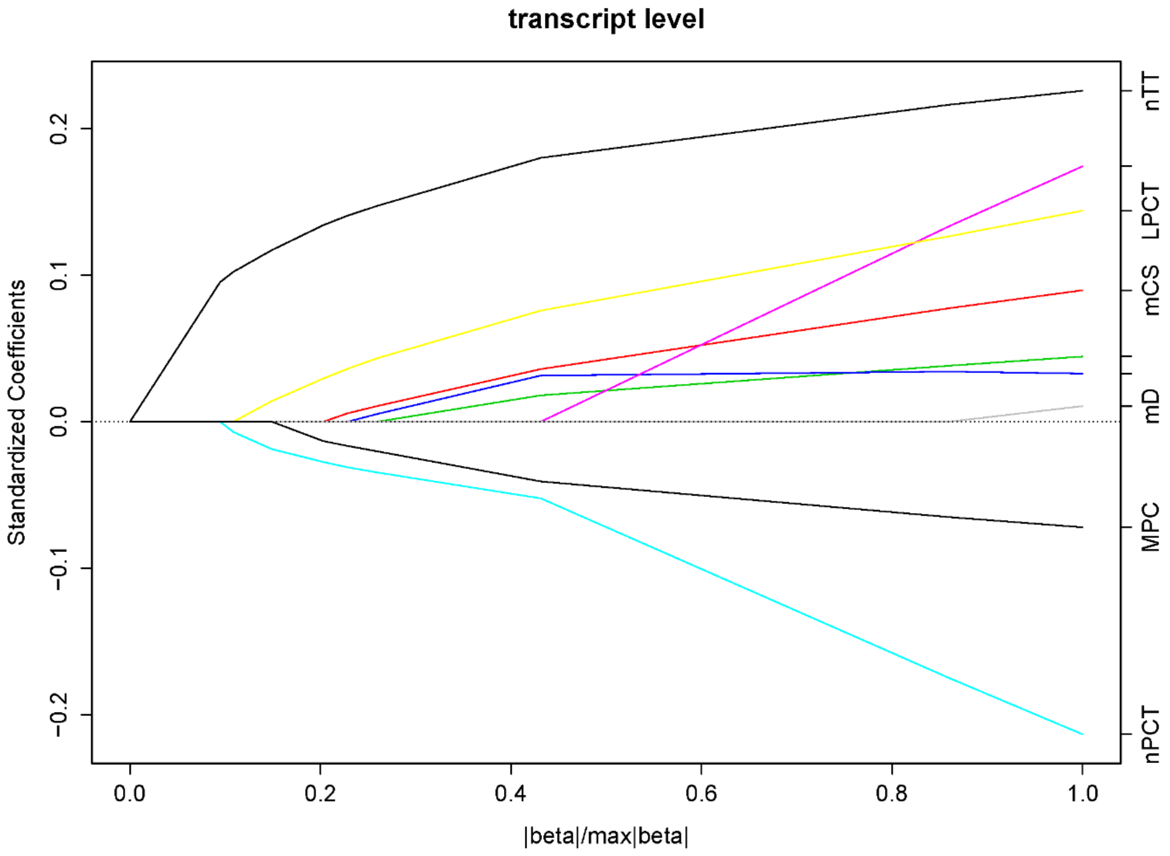

b)

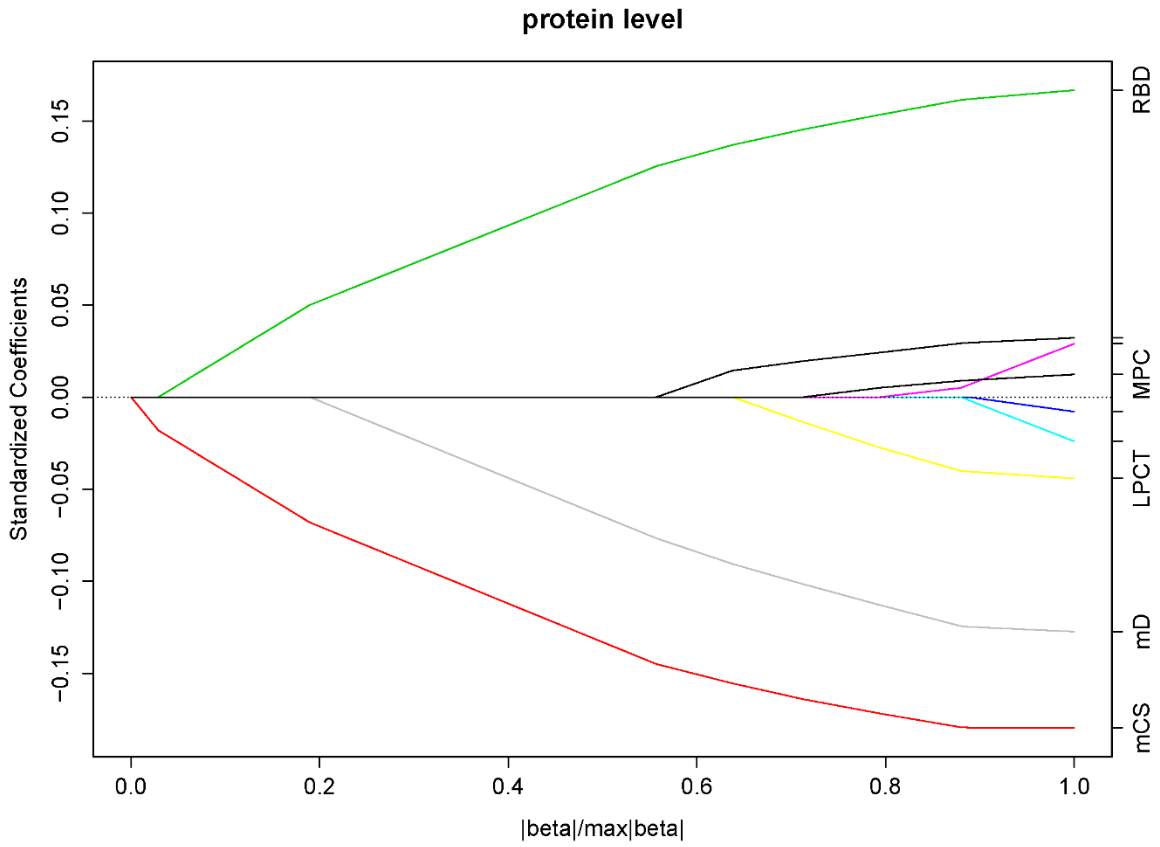

Supplementary figure 5

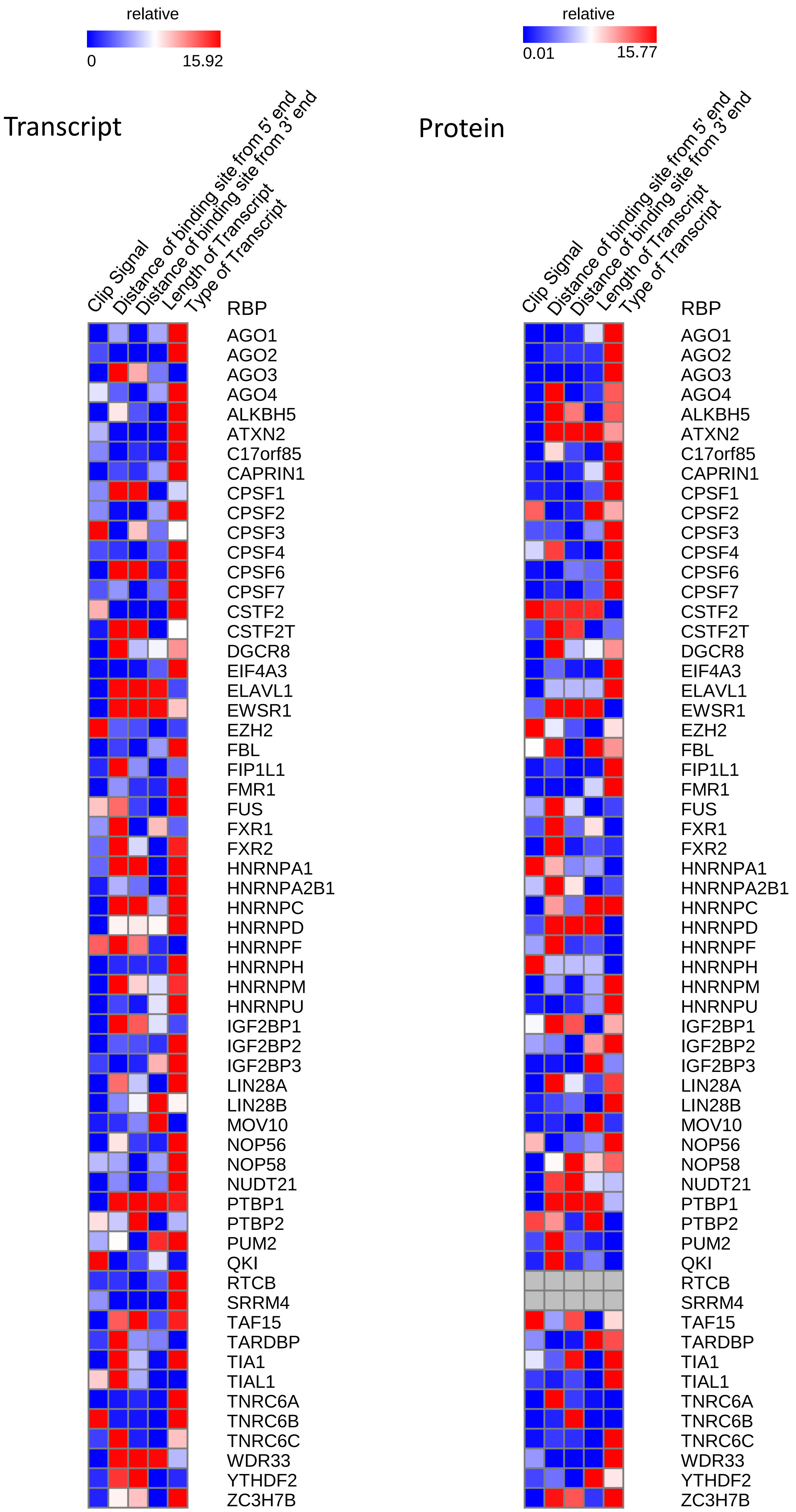

Supplementary Table 1

BioGrid:

SYNC RBPs				
**RBPs**	**Degree**	**Clustering Coefficient**	**Betweenness**	**Closeness**
AGO1	44	0.02	60472.139	0.00025
AGO3	68	0.005	81447.062	0.0002
CPSF1	57	0.016	59141.367	0.00025
CPSF4	18	0.072	4841.104	0.00019
CPSF6	83	0.008	55782.235	0.0002
CPSF7	74	0.014	79493.614	0.00025
FMR1	55	0	63287.892	0.0002
HNRNPC	168	0.006	139124.034	0.00023
HNRNPU	519	0.001	606967.202	0.00025
MOV10	994	0	1148344.935	0.00033
PTBP2	22	0.026	13595.795	0.0002
PUM2	16	0	12493.691	0.00019

NON-SYNC RBPs				
**RBPs**	**Degree**	**Clustering Coefficient**	**Betweenness**	**Closeness**
AGO2	90	0.005	118926.942	0.00016
AGO4	67	0.004	54735.439	0.00015
ALKBH5	4	0	188.434	0.00013
ATXN2	56	0.012	54610.188	0.00016
C17ORF85	39	0	29008.895	0.00015
CAPRIN1	39	0.043	15960.984	0.00016
CPSF2	48	0.044	39521.259	0.00015
CPSF3	42	0.053	23114.966	0.00015
CSTF2	75	0.023	82978.778	0.00017
CSTF2T	20	0.168	10889.791	0.00016
DGCR8	51	0.019	60836.47	0.00017
EIF4A3	258	0.003	273983.579	0.00017
EWSR1	649	0.001	1024972.094	0.00021
FBL	250	0.006	298531.117	0.00017
FIP1L1	58	0.031	45743.06	0.00015
FUS	361	0.006	507767.513	0.00021
FXR1	72	0.01	74495.727	0.00015

FXR2	122	0.007	181205.292	0.00017
HNRNPD	276	0.005	354840.867	0.00019
HNRNPH	95	0.018	81394.104	0.00017
HNRNPM	252	0.009	244242.176	0.00018
IGF2BP1	71	0.006	38707.81	0.00015
IGF2BP2	33	0.064	24985.179	0.00016
LIN28A	90	0.004	87765.186	0.00015
NOP58	110	0.017	77466.239	0.00016
NUDT21	120	0.004	146965.559	0.00016
PTBP1	139	0.019	120213.074	0.00018
TARDBP	327	0.007	407768.294	0.00019
TIA1	38	0	36107.231	0.00015
TIAL1	57	0	50972.762	0.00015
YTHDF2	40	0.029	24319.372	0.00016

STRING:

SYNC RBPs				
**RBPs**	**Degree**	**Clustering Coefficient**	**Betweenness**	**Closeness**
AGO1	61	0.046	8200.237	0.00139
AGO3	55	0.056	3164.624	0.00128
CPSF1	10	0.289	40.221	0.001
CPSF4	32	0.05	4819.317	0.00116
CPSF6	20	0.105	2578.936	0.00131
CPSF7	104	0.022	11591.343	0.0016
FMR1	49	0.006	10534.999	0.00134
HNRNPC	10	0	135.495	0.00103
HNRNPU	119	0.014	16270.507	0.00159
MOV10	44	0.066	4732.135	0.00128
PTBP2	33	0	7691.077	0.00122
PUM2	21	0	4557.498	0.00118

SYNC RBPs				
**RBPs**	**Degree**	**Clustering Coefficient**	**Betweenness**	**Closeness**
AGO2	65	0.025	31779.344	0.00047

AGO4	56	0.027	19548.296	0.00042
ALKBH5	5	0	3246	0.00029
ATXN2	24	0.014	10031.608	0.00044
C17ORF85	10	0	5679.216	0.00035
CAPRIN1	9	0.083	1320.282	0.00041
CPSF2	159	0.061	21813.829	0.00049
CPSF3	157	0.062	22021.022	0.00049
CSTF2	136	0.077	14883.491	0.00049
CSTF2T	29	1.11E-01	1553.181	0.00043
DGCR8	31	0.032	7537.98	0.00042
EIF4A3	182	0.004	102746.524	0.00054
EWSR1	32	0.018	13113.795	0.00044
FBL	183	0.01	80217.904	0.00052
FIP1L1	34	0.13	3359.221	0.00036
FUS	127	0.085	20844.026	0.0005
FXR1	39	0.051	12272.238	0.00044
FXR2	37	0.056	10620.347	0.00044
HNRNPD	155	0.057	41603.681	0.00051
HNRNPM	125	0.09	23873.586	0.00052
IGF2BP1	35	0.008	14773.88	0.00043
IGF2BP2	26	0.009	10506.682	0.00042
LIN28A	26	0.006	13431.744	0.00038
NOP58	164	0.009	52877.228	0.00047
NUDT21	110	0.112	4562.836	0.00048
PTBP1	140	0.068	27709.284	0.0005
TARDBP	37	0.018	16127.525	0.00044
TIA1	17	0	8644.176	0.00036
TIAL1	15	0	8587.596	0.00041
YTHDF2	4	0	2436	0.0004
